# Smoothelin-like protein 1 promotes insulin sensitivity and modulates the contractile properties of endometrial epithelial cells with insulin resistance

**DOI:** 10.3389/fendo.2024.1375771

**Published:** 2024-05-31

**Authors:** Ilka Keller, Ádám Ungvári, Richárd Kinter, Fanni Szalmás, Endre Kókai, Beáta Lontay

**Affiliations:** Department of Medical Chemistry, Faculty of Medicine, University of Debrecen, Debrecen, Hungary

**Keywords:** endometrium, insulin resistance, insulin signaling, migration, gene expression

## Abstract

**Introduction:**

The incidence of infertility is significantly higher in women with diseases linked to impaired glucose homeostasis, such as insulin resistance. Defective glucose metabolism interferes with fertilization; however, the molecular mechanism underlying this interference is unclear. Smoothelin-like protein 1 (SMTNL1) was isolated from muscle and steroid hormone-responsive tissues and regulates the contractile functions of various cell types through the inhibition of myosin phosphatase (MP) holoenzyme. In addition, SMTNL-1 after phosphorylation at Ser301 by protein kinase A translocates to the nucleus and functions as a transcriptional co-activator of the progesterone receptor-B. SMTNL1 null mice exhibit reduced reproductive fitness and are more prone to type 2 diabetes mellitus. However, the role of SMTNL1 in endometrial epithelial cells is not known.

**Methods:**

The effect of SMTNL1 overexpression was investigated in pregnancy and in gestational diabetic endometrial epithelial cell models by immunofluorescent staining, cell migration, and semi quantitative Western blot analysis and glucose uptake assay.

**Results:**

We show that SMTNL1 promotes the differentiation of endometrial epithelial cells in a progesterone-dependent manner to attenuate insulin resistance. Furthermore, SMTNL1 hampers the migration capacity of epithelial cells in a gestational diabetes model by inhibiting the expression of MYPT1, the regulatory subunit of MP, and the activity of the holoenzyme, resulting in increased phosphorylation of the 20 kDa regulatory myosin light chain. SMTNL1 also acts as an insulin-sensitizing agent by increasing the gene expression of PP2A and DUPS9 protein phosphatases, resulting in decreased ERK1/2 activity and, hence, decreasing the phosphorylation of IRS-1 at Ser612 under gestational diabetes conditions.

**Conclusion:**

SMTNL1 may have therapeutic relevance to the progesterone-dependent inhibition of endometrial epithelial cell migration under hyperglycemic conditions and insulin sensitivity in the endometrium in gestational diabetes or other metabolic disorders.

## Highlights

- SMTNL1 induces differentiation of endometrial epithelial cells
- SMTNL1 hampers migration of epithelial cells in a gestational diabetes model
- SMTNL1 promotes insulin sensitivity in insulin-resistant endometrial epithelial cells by decreasing IRS Ser612 phosphorylation
- SMTNL1 regulates the expression of PP2A and DUSP9, which regulate ERK1/2
- SMTNL1 increases glucose uptake and glycogen storage

## 1 Introduction

Morphological changes in the uterine epithelium are essential for the female reproductive cycle and the process of implantation. Secretory cells in the uterine epithelium undergo morphological changes during the luteal phase of the female cycle. The expression of cell surface molecules such as Mucin-1 (MUC-1) indicates successful differentiation and receptive endometrium (1). Aberrations in the single-layered columnar epithelium and secretory cells lead to inadequate trophoblast-to-endometrium communication, leading to implantation failure before cell-cell interaction can occur. Imbalances in steroid hormones and other pathological processes such as insulin resistance and the resulting metabolic syndrome can cause severe deformities, differentiation disorders, and pathological changes in the integrity of the endometrial epithelium (2). These defects are associated with various infertility disorders such as polycystic ovarian syndrome and may be the underlying cause of several aberrances leading to oligo-anovulation (3). Defective ovulation, sexual steroid hormone homeostasis, or blastocyst implantation induce a variety of clinical symptoms, including infertility. Infertility occurs in 5–10% of the reproductive-age female population (4). Additionally, high insulin and glucose levels and stimuli promoting epithelial cell proliferation and differentiation increase the risks of endometriosis and endometrial hyperplasia leading to endometrial cancer (5–7).

The underlying molecular mechanism of insulin resistance is multifactorial and includes the depletion of cell surface receptors, loss of function mutations, and unbalanced posttranslational modifications of molecules in the insulin receptor signaling pathway, including phosphorylation, enhanced Ser phosphorylation and reduced Tyr phosphorylation by Ser/Thr protein kinases (8). Serine phosphorylation of the insulin receptor substrate 1 (IRS-1) results in inhibition of its binding to the insulin receptor and tyrosine phosphorylation, therefore inhibition of binding of the downstream phosphatidyl-inosotol-3-kinase (PI3K) and activation of protein kinase B/Akt (9). Akt binds to GLUT4-containing vesicles and mediates insulin-stimulated glucose transport (10).

SMTNL1 is expressed in rabbit ileum (11), steroid hormone-responsive tissues, smooth muscle, and skeletal muscle (12). In addition, SMTNL1 regulates contractile functions in smooth muscle cells by inhibiting MP holoenzyme via binding to the MYPT1 regulatory subunit of MP, resulting in elevated phosphorylation of the 20 kDa regulatory subunit (MLC20) (12). Furthermore, phosphorylation on SMTNL1 Ser301 by protein kinase A and G results in SMTNL1 translocation to the nucleus where it functions as a transcriptional coregulator of progesterone receptor (PR) to regulate the expression of cytoskeletal elements, steroid receptors, and metabolic enzymes (13).

SMTNL1 is a potent regulator of insulin signaling, as shown in an *in vitro* insulin-resistant C2C12 rodent cell line skeletal muscle model. In mouse and differentiated skeletal muscle cell culture models of insulin resistance, SMTNL1 can attenuate the effects of altered IRS-1 serine phosphorylation induced by the over-activation of Ser/Thr protein kinases (14, 15). By reducing the expression of novel type PKCϵ (nPKCϵ), SMTNL1 can downregulate the activity of ERK 1/2 MAPK, resulting in decreased IRS-1 Ser612 phosphorylation, which was boosted by *in vitro* hyperinsulinemic-hyperglycemic conditions (14, 16). Thus, SMTNL1 prevents the dissociation of the IRS-1-PI3K-Akt-mTOR pathway and acts as an insulin-sensitizing agent in our *in vitro* rodent model. Moreover, SMTNL1 can induce GLUT4-mediated glucose uptake in C2C12 cells (14). In *Smtnl1 ^-^/^-^* mice, fitness is reduced, including reduced litter size, elongated times between pregnancies, and glucose intolerance, especially in pregnant animals (15). Interestingly, endometrial prolactin (PRL) levels are reduced in late pregnancy in *smtnl1 ^-^/^-^* mice (12). Although the insulin-sensitizing role of SMTNL1 has been characterized in skeletal muscle, the molecular explanation of the above-mentioned SMTNL1 KO reproductive phenotype and the correlation of it with the insulin-resistant conditions was not yet known, nor was its effect on endometrial epithelial cells. The aim of this study was to determine the effects of SMTNL1 on insulin sensitivity and the contractile properties of human endometrial adenocarcinoma cells under pregnant and hyperglycemic/hyperinsulinemic conditions.

## 2 Materials and methods

### 2.1 Chemicals

All chemicals were obtained from Sigma-Aldrich (St. Louis, MO, USA) unless indicated otherwise.

### 2.2 Antibodies

All antibodies are listed in **Supplementary Table S2**.

### 2.3 Cell culture and maintenance of Ishikawa cells

The human endometrial adenocarcinoma Ishikawa cell line (ECACC, #99040201) was maintained in Dulbecco’s modified Eagle’s medium (DMEM) with phenol red supplemented with 1000 mg/L glucose, 5% fetal bovine serum (FBS), 2 mM L-glutamine (Lonza, Basel, Switzerland), and 1% non-essential amino acids (NEAA). Cells were grown in a humidified incubator in an atmosphere of 5% CO_2_ at 37°C and passaged every 3 days at a 3:1 ratio.

### 2.4 Transient transfection and differentiation

The full-length human SMTNL1 construct (pcDNA-3.1-M11-FT-SMTNL1 plasmid expression vector, referred to as FT-SMTNL1, GeneCopoeia Rockville, USA) and the empty pcDNA-3.1 plasmid as a transfection control (MOCK) were overexpressed in Ishikawa cells. Both the FT-SMTNL1 plasmid and the MOCK plasmid (used in 2.5x10–^3^ μg/μl concentrations) were supplemented by Gene Juice transfection reagent (4 μl per well) and were suspended in 400 μl/well serum-free DMEM, then incubated for 15 minutes. After incubation mixture was added to adhered cells, previously seeded on 6 well plates in 2x10^5^/well density, and then culture was incubated at room temperature for another 15 minutes. After incubation serum free DMEM was further completed by 900 μl of serum containing complete growth medium to reach 1500 μl/well final volume. Cells were cultured for 72 hours after transfection.

### 2.5 *In vitro* pregnancy and gestational diabetes model of Ishikawa cells

The day after culturing or transfection, cells were washed with 1x PBS and complete growth medium was replaced by DMEM without phenol red, supplemented with 1000 mg/L glucose, 5% FBS, 2 mM L-glutamine, and 1% NEAA. For the pregnancy model, the medium was supplemented with 1 µM medroxy-progesterone-acetate, 10 nM 17-β-oestradiol, and 0.5 mM 8-Br-cAMP to represent physiological hormones of gestation (P4). For the gestational diabetes group (GDB), P4 cells were supplemented with 100 nM insulin and 0.025 mM glucose. Cells were lysed 72 hours after treatment.

### 2.6 Viability assay

Cells were seeded and transfected as described above. Cultures were maintained for 72 hours, and then 20 µM/well AlamarBlue (Invitrogen™, Waltham, Massachusetts, USA) reagent was added. Plates were incubated at 37°C for 90 minutes or until color change was evident. Fluorescence was measured at 530/590 nm with a fluorimeter (Tecan Group Ltd, Switzerland). The fluorescence of blank samples was subtracted, and the results were normalized to the average of control values.

### 2.7 Cell migration assay

Transfected Ishikawa cells were labeled with Vybrant™ DiD Cell-Labeling Solution (Invitrogen™, Waltham, Massachusetts, USA, Catalog number: V22887), seeded into collagen-coated OptiPlate-96 Black plates (PerkinElmer; Waltham, MA, USA), and cultured for 48 hours under control, P4, or GDB conditions. Each well was scratched by a Tecan Freedom EVO 150 machine (Tecan Group Ltd, Switzerland), and then cultured for an additional 24 hours; migratory potential was measured during this period. Cells were monitored using real-time screening for 24 hours with a 10x air objective (NA: 1.15) using the Opera Phoenix™ High Content Confocal System (HCS) instrument (PerkinElmer; Waltham, MA, USA). Free surface areas and cell numbers were analyzed using the built-in Harmony software (version 4.8). Results were normalized to the average of MOCK samples.

### 2.8 Immunofluorescence staining

The confocal microscopy protocol was previously described (16). Briefly, the actin cytoskeleton was stained with Texas-Red Phalloidin (yellow) and nuclei were labeled with DAPI (4′, 6-diamidino-2-phenylindole) (blue). Anti-MUC-1, anti-MYPT1, anti-MYPT1^pT696^, anti-MLC, and anti-MLC^pS18^ antibodies were used at 1:100 dilutions and labeled with Alexa488 fluorophore in 1:1000 dilution (green). Visualization was carried out using the Opera Phoenix™ HCS. A total of 14–16 fields with 3x 10^4^ cells were acquired per well and laser-based autofocus was performed for each imaging position. Images of DAPI, Alexa-488 and Alexa-647 channels were collected at the 0 µm position of the Z image plane relative to the bottom of the optical plate using a 10x objective. Intensity of Alexa 488 fluorophore has been calculated as follows: Analysis have been conducted by measuring sum of Alexa 488 fluorophore intensity of 9 fields of each well, 3 independent wells per groups, by built in intelligent software of high content screening instrument. Analyzed intensity have been filtered to autofluorescence, and intensity of secondary antibody control was subtracted from each well’s sum intensity. Data was normalized to cell number obtained from nuclei number stained with DAPI. Control MOCK group value was taken as 100%, and all other groups were expressed as ratios.

### 2.9 Protein extraction

Protein extraction was conducted as previously described (17). Total protein concentrations were determined using the bicinchoninic acid (BCA) Protein Assay Kit (Thermo Scientific, Waltham, MA, USA).

### 2.10 Western blot analysis

Semi-quantitative Western blot analyses were conducted, as previously described (16) with modifications as follows. Protein (50 µg) was loaded onto 4–20% precast Criterion gel (Bio-Rad Laboratories) and separated at 200 V. Nitrocellulose membranes were blocked in 5% (w/v) bovine serum albumin/tris-buffered saline containing 0.1% (v/v) Tween 20 (BSA/TBST) for at least 1.5 hours. The membranes were incubated with primary and secondary antibodies diluted in blocking solution. Antibody binding was detected with enhanced chemiluminescence (WesternBright ECL or WesternBright Sirius and WesternBright Peroxide reagents, Advansta Inc.; San Jose, CA, USA) at a ratio of 1:1 in a ChemiDoc Touch Imaging System (Bio-Rad Laboratories; Hercules, CA, USA).

### 2.11 Glycogen content measurement

Cells were seeded on 6-well plates, grown to 70% confluency, and transfected with FT-SMTNL1 or MOCK vector. After 72 hours, cells were trypsinized for 5 mins; the reaction was terminated by adding 6 times volume of complete growth media. Cells were centrifuged for 4 minutes at 2000 g at 4°C. Pellets were solubilized in 0.1 M KH_2_PO_4_ followed by sonication at 10% output (Branson Sonifier 250. Fisher Scientific). After adding 25% TCA, the lysate was vortexed and centrifuged at 16000 g for 5 minutes at 4°C. After adding concentrated H_2_SO_4_, the supernatants were incubated for 30 minutes at room temperature. The mixture was incubated with 6% phenol for 5 minutes at 90°C. The glycogen content was measured based on absorbance at 490 nm, and concentration was calculated against standard curve, consisting of glycogen stock of the following concentrations: 0.00625 g/100 ml, 0.025 g/100 ml, 0.1 g/100 ml and reagent blank. Blank absorbance was subtracted from each wells value. Results of glycogen concentration in g/100 ml were normalized to protein concentration, measured by BCA assay from each sample, protein concentration expressed as g/100 ml unit. Normalized results had a unit of glycogen/protein (g/g). MOCK control group value was taken as 100%, and all other groups were expressed as ratios as bar chart.

### 2.12 Glucose uptake assay

The 2-NBDG (2-N-7-Nitrobenz-2-oxa-1,3-diazol-4-yl-Amino-2-Deoxyglucose) uptake was assessed as described previously (14). Ishikawa cells were cultured, transfected, treated as previously described, washed with PBS, and incubated in complete growth media (glucose-free DMEM) for 1 hour. After adding 100 μM 2-NBDG dissolved in complete growth media, cells were washed with PBS and lysed in 0.1 M KH_2_PO_4_ (pH 11.0) lysis buffer supplemented with 1% Triton X-100. Fluorescence was measured at an excitation of 485 nm and an emission of 535 nm using a Tecan Spark Multimode microplate reader (Tecan Treading AG, Mannedorf, Germany). Fluorescence was normalized to protein concentrations.

### 2.13 RNA isolation and semi-quantitative RT-PCR analysis

Total RNA was extracted from Ishikawa cell culture after P4 and GDB treatment with or without FT-SMTNL1. TRIzol reagent (1 ml, Life Technologies, Carlsbad, CA) was added to each well. Cell lysates were treated with 200 µl chloroform and centrifuged at 13000 rpm at 4°C for 15 minutes. Isopropanol (500 µl) was added to the RNA fraction, and the mixture was centrifuged at 13000 rpm at 4°C for 10 minutes. The pellet was resuspended in 75% ethanol and centrifuged again at 13000 rpm at 4°C for 5 minutes. The pellet was resuspended in 10 µl of nuclease-free water. Concentration was measured using a NanoDrop Fluorospectrometer (Thermo Fisher Scientific, Waltham, Massachusetts, USA). Reverse transcription was carried out using 0.6 µg RNA/sample at 37°C for 120 minutes using a cDNA Synthesis Kit (Thermo Fisher Scientific, Waltham, Massachusetts, USA). The cDNA was used for PCR amplification with a 2x Xceed qPCR SyberGreen Master mix (Institute of Applied Biotechnologies, Prague – Strašnice, Czech Republic). The PCR primers are listed in **Supplementary Table S1**. The PCR protocol is as follows: 95°C for 3 minutes for denaturation; 50 cycles of 95°C for 3 seconds, 60°C for 30 seconds, 72°C for 90 seconds. PCR was performed using a LightCycler 480 (Roche Applied Science, Penzberg, Germany) machine. The Cp values of each sample were normalized to the geometric mean of housekeeping genes, including GAPDH and Cyclophilin A. Data was normalized to the value of MOCK vector treatment.

### 2.14 Statistical analysis

Image J software was used to quantify Western blot images. Data were plotted as bar charts and statistical analyses were calculated using GraphPad Prism 8 software. Phosphorylated proteins were normalized to non-phosphorylated protein expression, while non-phosphorylated protein expression was normalized to loading controls. All groups were normalized to the MOCK vector, which was set at 100%. Data are presented as mean ± SD for n = 3–6 independent experiments. Differences were considered statistically significant at p < 0.05 using unpaired two-tailed t-tests to compare two groups.

## 3 Results

### 3.1 SMTNL1 overexpression facilitates the differentiation of endometrial epithelial cells

**Figure 1:**
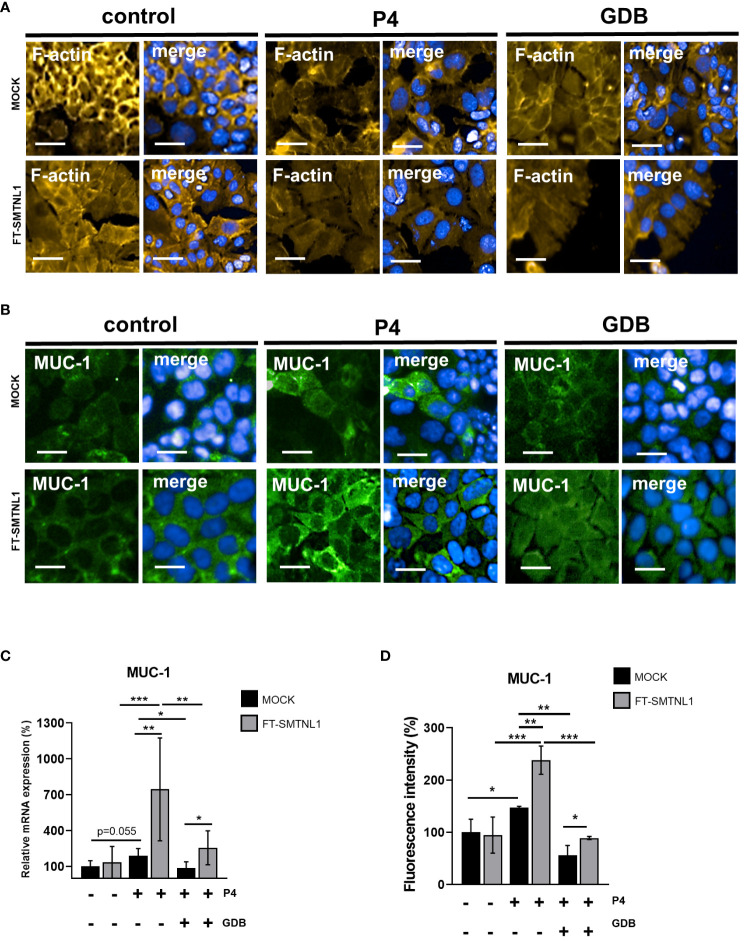
SMTNL1 overexpression facilitates the differentiation of Ishikawa cells. Differentiation was assessed by staining of F-actin of Ishikawa cells using Texas-Red Phalloidin dye (yellow) in control/P4/GDB groups. Nuclei were stained with DAPI (blue). Scale bars: 100 μm **(A)**. Differentiation of Ishikawa cells in control/P4/GDB groups was followed by high-content microscopy by staining for the MUC-1 differentiation marker with Alexa 488 fluorophore (green). Nuclei were stained by DAPI (blue). Scale bars: 100 μm **(B)**. MUC-1 differentiation marker mRNA expression was followed by RT-PCR. Cp values were normalized to housekeeping genes GAPDH and Cyclophilin A and were expressed as ratios of untreated MOCK control taken as 100%. Data was plotted as bar charts, where values represent n=3–8 +/- SD **(C)**. Muc-1 protein fluorescent intensity was assessed by HCS system **(D)** Two groups were compared by unpaired two-tailed t-test, and four groups or more were compared by two-way ANOVA where: p< 0.05 (*), p<0.01 (**) and p<0.0001 (***) **(C, D)**. P4: progesterone, GDB: insulin resistance with P4, gestational diabetes.

SMTNL1 acts as a key regulator in steroid hormone-sensitive tissues such as the uterus; however, the detailed mechanism is unclear. To assess the effect of SMTNL1 on cell differentiation and cell viability, Ishikawa endometrial epithelial cells were transfected with the empty vector (MOCK control) or the FT-SMTNL1 plasmid. In our experiments, we used Ishikawa cell line modelling the inner columnar epithelium of the endometrium, which differs in function and structure from the underlying endometrial stromal cells (18). Validation of overexpression was conducted by immunofluorescence (**Supplementary Figures S1A, C, D, F**) and Western blot analysis (**Supplementary Figure S1B**) applying anti-Flag (**Supplementary Figures S1A–C**) and anti-SMTNL1 antibodies (**Supplementary Figures S1D–F**). Immunofluorescence staining with the Flag-tag antibody showed cytosolic localization of SMTNL1 expression with lower nuclear SMTNL1 expression, staining with anti-SMTNL1 antibody resulted in same peripherial, homogenous arrangement (**Supplementary Figure S1D**). Analysis of Alexa 488 intensity labelling anti-Flag-tag antibody shown an elevation of 171% (p=0.012) comparing FT-SMTNL1 overexpressed group to MOCK control, and elevation by 165% (p=0.0141) compared with untreated control group (**Supplementary Figure S1C**). SMTNL1 fluorescence intensity was elevated by 128.67% (p=0.0025) and by 150% (p=0.0055) in FT-SMTNL1 overexpressed group compared to control and MOCK (**Supplementary Figure S1F**). Investigating the endogenous SMTNL1 expression in the applied control, P4 and GDB environments a decrement of expression was seen in GDB group, by 56.75% (p=0.034) and by 48% (p=0.0290) compared with control and P4 groups, respectively (**Supplementary Figure S1E**). FT-SMTNL1 expression increased by 177% (p = 0.0002) and 191.5% (p<0.0001) in cells transfected with FT-SMTNL1 compared with control (untreated control) and MOCK group, respectively (**Supplementary Figure S1B**). FT-SMTNL1 transfection in Ishikawa cells did not affect cell viability quantified by Alamar Blue assay (**Supplementary Figure S1G**). The effects of SMTNL1 on differentiation of Ishikawa cells were assessed by HCS screening microscopy and RT-PCR. To investigate morphological changes in Ishikawa cells, the actin cytoskeleton was labeled with Texas Red phalloidin (yellow) and MUC-1 differentiation marker was labeled with Alexa 488 fluorophore (green) (**Figures 1A, B**). The mRNA expression of MUC-1 was assessed by RT-PCR (**Figure 1C**). Ischikawa cells were subjected to progesterone treatment (P4) and hyperglycaemic/hyperinsulinaemic treatment (GDB) modeling insulin resistance in the presence of progesterone mimicking disorders such as gestational diabetes based on the protocols of previous publications (19, 20). These results are summarized in **Supplementary Figure S2**. showing that the expression levels of Akt remained unchanged (**Supplementary Figure S2A**). Although Akt^pS473^ phosphorylation was increased in the control group upon acute insulin treatment (**Supplementary Figure S2B**) but there was no increase in Akt phosphorylation in the GDB samples proving the insulin resistant state of the epithelial cells. P4 and GDB treatments induced cytoskeletal changes; the undifferentiated cuboidal morphology shifted towards an elongated, columnar shape. SMTNL1 overexpression further promoted these changes in a non-progesterone-dependent manner (**Figures 1A, B**). The synergic effects of SMTNL1 and progesterone were indicated in the P4 and GDB groups as prominent morphological changes. MUC-1 expression was evoked by both SMNTL1 and progesterone. SMTNL1 overexpression enhanced MUC-1 intensity the most in P4 environment, to which decrement was detectable in control FT-SMTNL1 overexpressed group by 143.33% (p<0.0001) and by 149% (p<0.0001) in GDB environment overexpressed group. The MUC-1 differentiation and mRNA expression were most prominent in the P4 SMTNL1-overexpressed group (**Figures 1B–D**), suggesting the positive effect of SMTNL1 on the differentiation of endometrial epithelial cells in the presence of P4. SMTNL1 overexpression increased MUC-1 mRNA expression levels by 554.67% (p=0.0036) in the P4 SMTNL1 supplemented group, compared to the P4 MOCK control. SMNTL1 enhanced MUC-1 mRNA expression by 170% (p=0.026) in the GBD-transfected group compared to the GDB MOCK control. The expression of MUC-1 was increased by 90.67% (p=0.0551) in P4 MOCK cells compared with MUC-1 expression in the control MOCK group, indicating differentiation of Ishikawa cells in the P4 environment. In the GDB environment, MUC-1 mRNA expression decreased by 104.16% (p=0.0307) compared to P4 MOCK group, indicating that the hyperinsulinemic-hyperglycemic environment hampers differentiation. Comparing the SMTNL1 overexpressed groups the P4 treated environment evoked the highest expression of MUC-1 mRNA, which was decreased by 611.958% (p=0.0001) in control, and by 488.83% (p=0.0064) in GDB samples with FT-SMTNL1 overexpression (**Figure 1C**). Analyzing immunofluorescence images MUC-1 intensity labelled with Alexa 488 fluorophore elevated significantly in P4 MOCK group by 47.33% (p=0.0315) compared with untreated MOCK control. SMTNL1 overexpression elevated MUC-1 intensity by 90.67% (p=0.0031) and by 33% (p=0.0391) compared with P4 and GDB MOCK groups, respectively. GDB treatment hindered MUC-1 intensity by 91.33% (p=0.0029) compared to P4 MOCK samples (**Figure 1D**).

### 3.2 SMTNL1 overexpression decreases MYPT1 expression and inhibits MP activity

**Figure 2:**
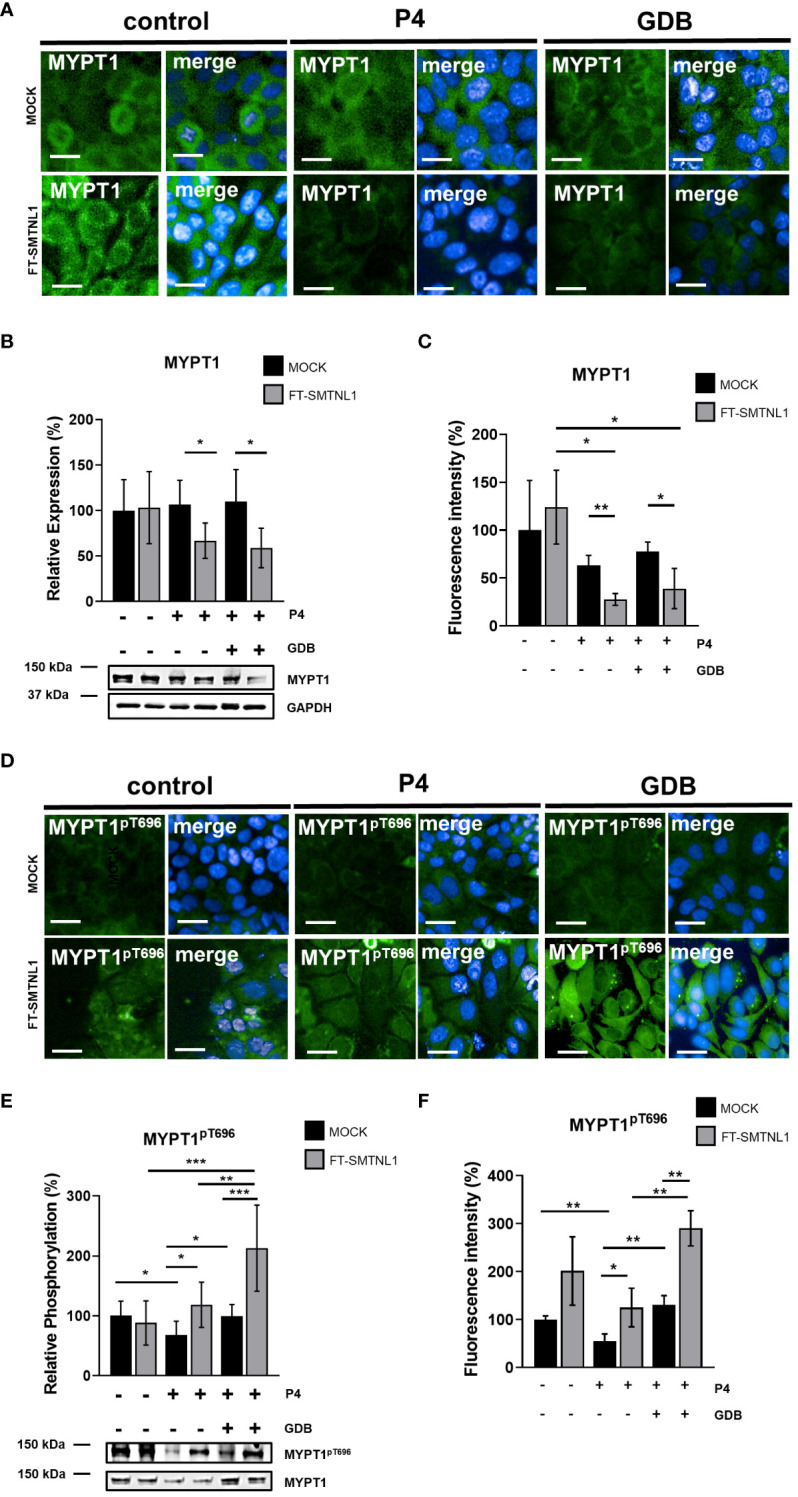
SMTNL1 inhibits the expression and the activity of MP in GDP in endometrial epithelial cells. Ishikawa cells were transfected with empty vector (MOCK) or FT-SMTNL1 in each of the control/P4/GDB treatments. Immunofluorescent staining was conducted using antibodies against MYPT1 **(A)** and MYPT1^pT696^ **(D)**, crosslinked with Alexa 488 fluorophore (green), nuclei were stained by DAPI (blue). Images were captured by Opera Phoenix™ HCS. Scale bar: 20µm. **(A, D)** The intensity of Alexa 488 Fluorophore labelling anti-MYPT1 **(C)** and anti-MYPT1p^T696^ **(F)** primary antibodies have been measured, by built in intelligent software of high content screening instrument. Data was normalized to cell number obtained from nuclei number stained with DAPI. Protein samples from whole-cell lysates were analyzed using Western blot analysis with anti-MYPT1 **(B)**, anti-MYPT1^pT696^ **(E)** antibodies. Values represent n =5–8, mean ± SD. Data represents the ratio to control MOCK group, which was considered to be 100%. Two groups were compared by unpaired two-tailed t-test, and four groups or more were compared by two-way ANOVA, where: p< 0.05 (*), p<0.01 (**) and p<0.0001 (***).

Changes in the expression of cytoskeletal proteins and their modifying enzymes contribute to changes in cell shape and differentiation. Therefore, the effects of SMTNL1 on its major interacting partner, MYPT1, were investigated (12, 13) (**Figure 2**). Ishikawa cells were cultured in control environment, in control environment further supplemented with P4 (P4), and P4 with the addition of high glucose and insulin (pathological GDB). MYPT1 activity was measured by quantifying the inhibitory phosphorylation site in Ishikawa cells after control, P4 and GDB treatment for 72 hours, with or without FT-SMTNL1 overexpression. Immunofluorescent staining (**Figure 2A**) and its image analysis (**Figure 2C**) shows reduction in MYPT1 expression based on fluorescence intensity in both P4 an GDB groups upon FT-SMTNL1 overexpression by 35.34% (p=0.0079) and by 38.66% (p=0.0445) in P4 in GDB groups, respectively compared to their untransfected MOCK controls. The FT-SMTNL1 overexpression resulted in a 96.33% (p=0.0132) and a 85% (p=0.0301) decrease in P4 and GDB treatments, respectively compared to non-treated FT-SMTNL1 overexpressed group (**Figure 2C**). Changes in MYPT1 expression were also assessed by Western blot analysis and the results aligned with the HCS analysis data (**Figures 2A, C**). SMTNL1 overexpression reduced MYPT1 expression in the P4 and GDB groups by 39.49% (p=0.0187) and 50.91% (p=0.0175), respectively, compared to P4 MOCK and GDB MOCK groups, respectively (**Figure 2B**). These findings correlate with previously characterized ability of SMTNL1 to reduce MYPT1 expression in smooth and skeletal muscle as the coactivator of PR (12). Assessing MYPT1 activity, by measuring its inhibitory phosphorylation, SMTNL1 overexpression significantly increased MYPT1^pT696^ inhibitory phosphorylation in the GDB group by 113.66% (p=0.0002), compared to GDB MOCK. The inhibitory phosphorylation on MYPT1^pT696^ was decreased by 31.8% (p=0.0406) implementing P4 MOCK treatment compared to the control MOCK group, indicating MYPT1 activation in P4 environment, which was further hindered by FT-SMTNL1 transfection. It also elevated MYPT1 inhibitory phosphorylation by 50.3% (p=0.0286) in P4 FT-SMTNL1 treated group (**Figure 2E**). GDB treatment caused inactivation of MYPT1 by elevating Thr696 phosphorylation by 30.94% (p=0.0304) compared to P4 MOCK group, which inactivation was further increased upon FT-SMTNL1 overexpression by 113.66% (p=0.0002) in SMTNL1 overexpressed GDB group. SMTNL1 overexpression evoked the most potent inhibition of MYPT1 in GDB environment, compared to which decrement was detectable in control SMTNL1 overexpressed group by 124.675% (p<0.0001), and by 94.3% (p=0.036) in P4 treated SMTNL1 overexpressed group. Western Blot analysis data were supported by immunofluorescent staining where nuclei were labelled by DAPI (blue), MYPT1^pT696^ was visualized using Alexa 488 fluorophore (green) (**Figure 2D**). Analyzing the intensity of MYPT1^pT696^ labelled with Alexa 488, inhibitory phosphorylation of MYPT1 was elevated by FT-SMTNL1 overexpression by 70% (p=0.0473) in P4 and by 159.67% (p=0.0026) in GDB groups. Applying P4 treatment has reduced MYPT1 inhibitory phosphorylation by 45% (p=0.0098) compared with control MOCK group, and GDB environment resulted in an increase of phosphorylation by 75.66% (p=0.0059) compared to P4 MOCK group (**Figure 2F**). Our data verified the regulatory effects of SMTNL1 on MYPT1 expression and activity in Ishikawa cells. The cytoplasmatic and nuclear localization of MYPT1 did not change in the P4 and GDB groups.

### 3.3 SMTNL1 overexpression regulates MLC20 phosphorylation via inhibition of MP

**Figure 3:**
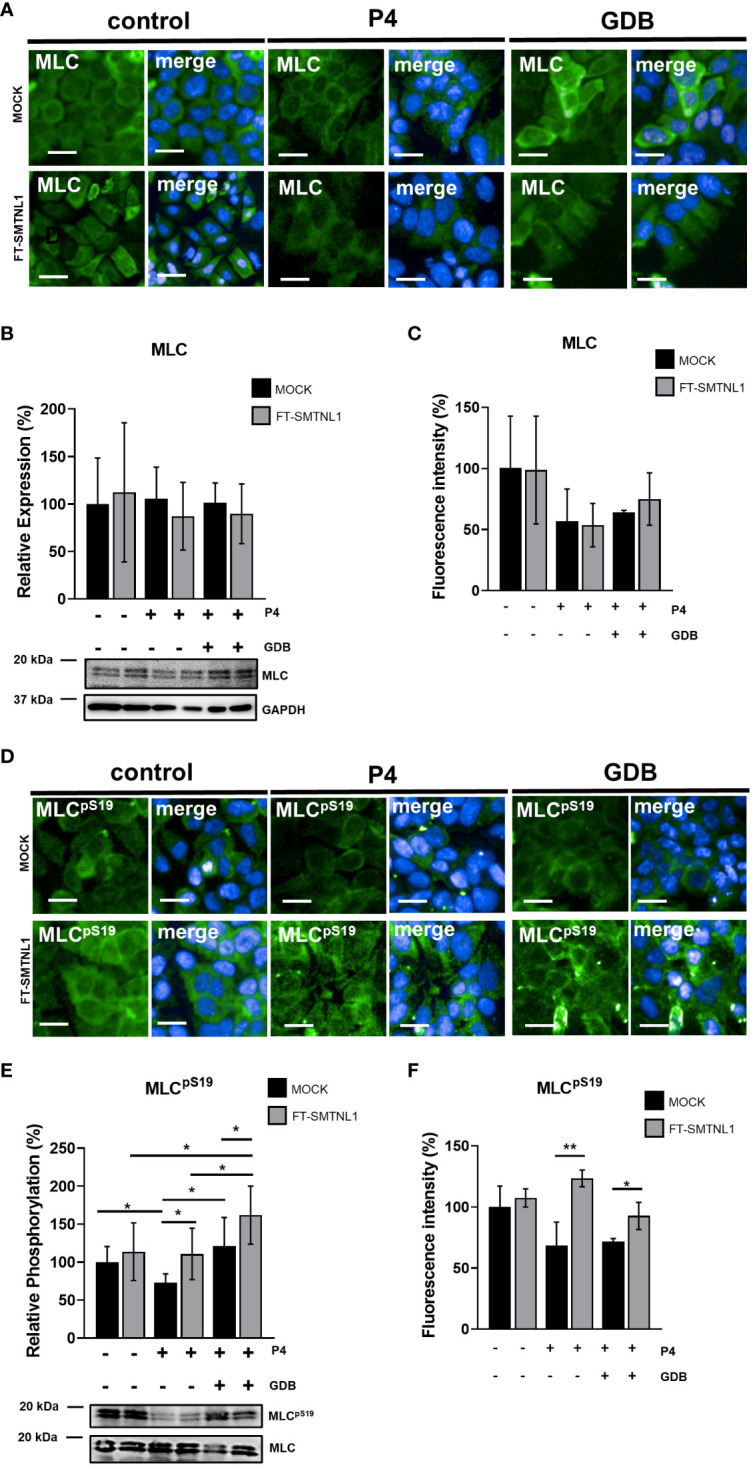
Effect of SMTNL1 on the regulatory 20kDa myosin light chain phosphorylation. Ishikawa cells were transfected with empty vector (MOCK) or FT-SMTNL1 in each of control, progesterone (P4) and insulin resistant (gestational diabetes; GDB) treatments. Immunofluorescent staining was conducted using antibodies against anti-MLC **(A)** and anti-MLCpS19 **(D)** detected with Alexa 488 fluorophore (green). Nuclei were stained by DAPI (blue). Pictures were captured by Opera Phoenix™ high-content screening microscopy. Scale bar: 20 µm. The intensity of Alexa 488 Fluorophore labelling anti-MLC **(C)** and anti-MLCp^S19^ **(F)** primary antibodies have been measured, by built in intelligent software of high content screening instrument. Data was normalized to cell number obtained from nuclei number stained with DAPI. Protein samples from whole-cell lysates were analyzed using Western blot with anti-MLC **(B)**, anti-MLCpS19 antibodies **(E)**. Values represent n =5–8, mean ± SD. Data represents the ratio of control MOCK group taken as 100%. Two groups were compared by unpaired two-tailed t-test, and four groups or more were compared by two-way ANOVA, where: p< 0.05 (*) and p<0.01 (**).

The overexpression of SMTNL1 reduced MYPT1 expression level in both P4 and GDB cells (**Figure 2**) and influenced MP activity via inhibitory phosphorylation in the GDB group (**Figure 2**). Because of these facts, we assessed the effect of SMTNL1 overexpression on the substrate of MP, the 20 kDa myosin light chain (MLC20). Neither the hormone treatments nor the SMTNL1 overexpression evoked any significant effects on MLC20 expression (**Figures 3A–C**). MLC expression and phosphorylation were measured by HCS microscopy after labeling nuclei with DAPI and MLC and MLC20^pS19^ with Alexa 488 fluorophore (green) (**Figures 3A, D**). Analyzing the intensity of Alexa488 fluorophore MLC has shown no changes in expression in any of the applied treatments (**Figure 3C**), and FT-SMTNL1 overexpression elevated the phosphorylation of MLC on the Ser19 residue by 31% (p=0.0014) in P4 and by 9% (p=0.0327) in GDB group compared with their untreated MOCK control (**Figure 3F**). MLC was predominantly localized in the cytoplasm in Ishikawa cells, and neither its localization nor expression changed in any of the experimental groups. MLC20^pS19^ phosphorylation was elevated in response to SMTNL1 overexpression in both the P4 and GDB groups, presumably due to the inhibition of MP. The decreased MYPT1 expression and elevated inhibitory phosphorylation of MYPT1 in response to SMTNL1 overexpression was accompanied by an increase in the phosphorylation of MLC20 at the Ser19 residue (**Figure 3E**) exclusively in the progesterone-supplemented groups. MLC20^pS19^ phosphorylation increased by 37.78% (p=0.0342) in the P4 group and by 40.91% (p=0.0315) in the GDB group comparing the effects of SMTNL1 overexpression to their MOCK controls. Progesterone decreased MLC20^pS19^ phosphorylation by 27% (p=0.0305) compared with MLC20^pS19^ phosphorylation in the non-treated MOCK control group. Comparing P4 MOCK group to GDB MOCK a 47.89% (p=0.0187) elevation of MLC20^pS19^ phosphorylation was detectable. Comparing SMTNL1 overexpressed groups, MLC phosphorylation was the most elevated in GDB environment, to which a decrease was detectable compared to both P4 and control environments by 51.02% (p=0.0306), and by 48.1% (p=0.0406), respectively (**Figure 3E**).

### 3.4 SMTNL1 inhibits the migration of endometrial epithelial cells

**Figure 4:**
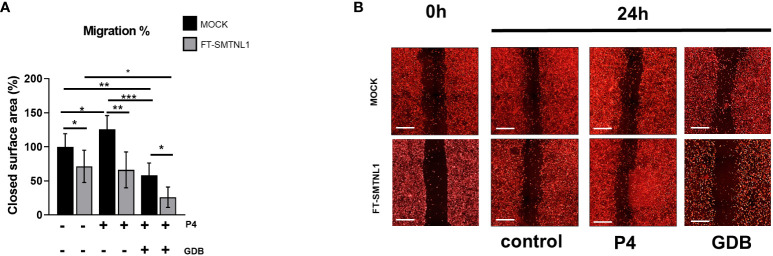
SMTNL1 hampers the migration capacity of endometrial epithelial cells. Scratch assay of DiD fluorescent dye-labeled Ishikawa cells transfected with either empty vector (MOCK) or FT-SMTNL1 and maintained in control, progesterone (P4), or gestational diabetes (GDB) environment. After 48 hours of treatment cells were scratched and were grown for an additional 24 h. Surface area of the closed territory was analyzed, and values were normalized and expressed as the ratio of the control MOCK group. Data was plotted as bar charts, where each column represents n=4-9, mean ± SD. Two groups were compared by unpaired two-tailed t-test, and four groups or more were compared by two-way ANOVA, where: p< 0.05 (*), p<0.01 (**) and p<0.0001 (***). **(A)** Photos of scratch areas were taken at the indicated times. Scale bars: 500 µm **(B)**.

SMTNL1 regulates MP regulatory subunit MYPT1 expression and activity on a progesterone-dependent manner in endometrial epithelial cells resulting in the altered regulatory phosphorylation of MLC20. Since MP and MLC20 are crucial key elements of the cellular contractile functions, affecting migration as well, we aimed to determine the effects of SMTNL1 overexpression on the migration of Ishikawa cells in the control, P4 and GDB groups (**Figure 4A**) using scratch assays. Changes in covered surface area were monitored for 24 h in real-time with HCS microscopy (**Figure 4B**). SMTNL1 overexpression in the control environment reduced the migration of Ishikawa cells by 28.67% (p=0.0227). The reduction of migration in response to SMTNL1 overexpression was more pronounced in the P4 and GDB groups by 59.68% (p=0.0003) and by 32.14% (p=0.0153), compared to the corresponding MOCK controls, respectively. P4 treatment increased the migration of Ishikawa cells by 25.8% (p=0.0355) compared to the MOCK-transfected cells, correlating with the natural induction of endometrial epithelial cell migration during pregnancy. Moreover, comparing FT-SMTNL1 overexpressed groups there was a 45.33% (p=0.0349) decrease between GDB and control treatments. However, GDB treatment reduced the migration of Ishikawa cells by 41.85% (p=0.0067), compared to untreated MOCK control, and by 67.66% (p<0.0001) compared to P4 MOCK group, indicating the pathological effects of impaired glucose homeostasis in gestational diabetes during hyperglycemia.

### 3.5 SMTNL1 regulates IRS-1 and Akt-1 Ser phosphorylation and activity in Ishikawa cells

**Figure 5:**
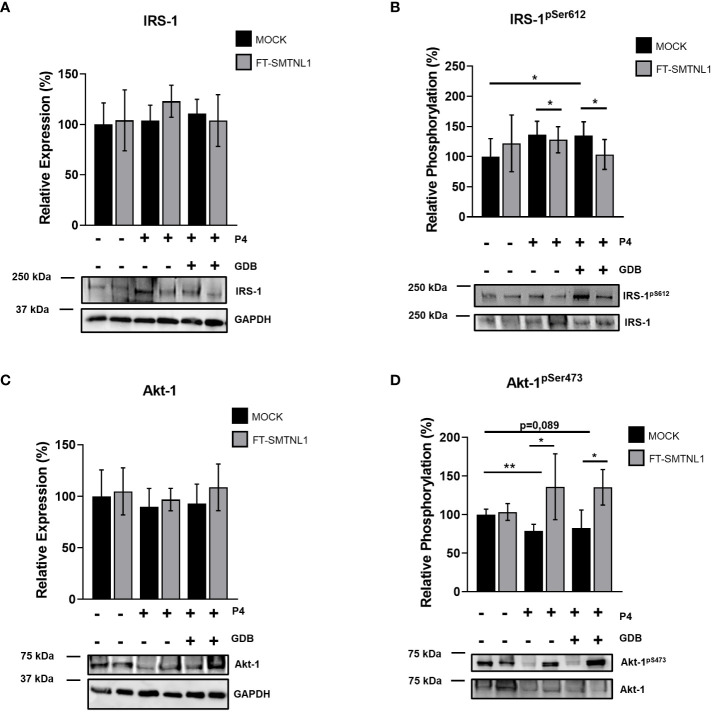
SMTNL1 overexpression affects the phosphorylation of insulin receptor substrate 1 and Akt-1 in Ishikawa cells. Empty vector (MOCK) or FT-SMTNL1-transfected Ishikawa cells were cultured under control, control pregnant (P4), or gestational diabetes (GDB) environments. Proteins were separated by SDS-PAGE followed by Western blot analysis using anti-IRS-1 **(A)**, anti-IRS-1pS612 **(B)**, anti-Akt-1 **(C)**, and anti-Akt-1pS473 antibodies **(D)**. Values represent n=3–8, mean +/- SD, expressed as the ratio of the control MOCK group. Two groups were compared by unpaired two-tailed t-test, and four groups or more were compared by two-way ANOVA, where: p< 0.05 (*) and p<0.01 (**).

Fertility disorders are often linked to insulin resistance. In addition, the incidences of type two diabetes mellitus and metabolic syndrome are higher in *smtnl1 ^-^/^-^ null* mice (12). A previous study suggests that SMTNL1 plays a crucial role in the regulation of insulin signaling in skeletal muscle (14). To elucidate the effect of SMTNL1 on insulin signaling in endometrial epithelial cells, we determined the protein and phosphorylation levels of IRS-1 and Akt-1 under P4 and GDB conditions (**Figure 5**). None of the treatments significantly affected the expression of IRS-1 (**Figure 5A**); however, IRS-1^pS612^ phosphorylation, the hallmark of insulin resistance, was elevated in MOCK-transfected cells in GDB groups by 34.87% (p=0.0203) compared with control conditions. SMTNL1 overexpression in combination with both P4 and GDB treatment decreased IRS-1^pS612^ phosphorylation compared with P4 and GDP MOCK cells by 23.62% (p=0.0228) and by 31.375% (p=0.0305), respectively (**Figure 5B**). In addition, Akt-1 expression was unaltered under any treatments (**Figure 5C**). P4 and GDB treatments decreased the phosphorylation of Akt-1^pS473^ by 21.33% (p=0.0034) and 17.66% (p=0.089), respectively, indicating the successful establishment of an insulin-resistant model (**Figure 5D**). Upon SMTNL1 overexpression, Akt-1^pS473^ phosphorylation was elevated by 57.33% (p=0.0110) and by 53% (p=0.0209) in the P4 and GBD groups, compared with the corresponding MOCK-transfected groups, respectively (**Figure 5D**). Collectively, these data demonstrated that pregnancy and hyperinsulinemia/hyperglycemia modeling gestational diabetes induced insulin resistance, and the overexpression of SMTNL1 attenuated this effect.

### 3.6 SMTNL1 regulates MAPK kinases and phosphatases to influence insulin resistance

**Figure 6:**
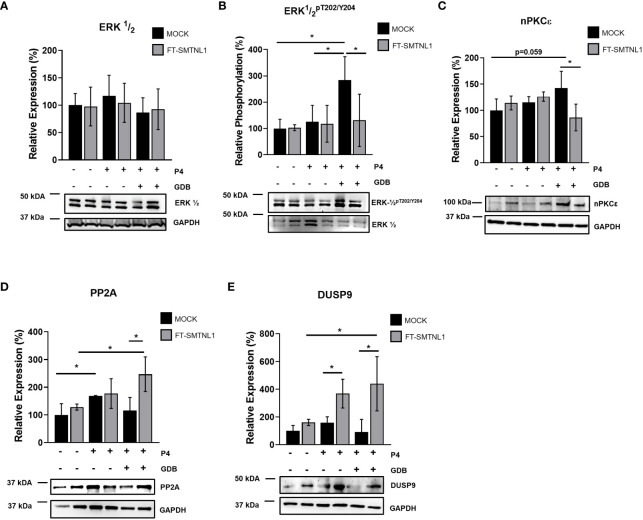
SMTNL1 overexpression affects ERK1/2 phosphorylation by regulating PP2A and DUSP9 expression in Ishikawa cells. Empty vector (MOCK) or FT- transfected Ishikawa cells were cultured in either control, control pregnant (P4), or gestational diabetes (GDB) environments. Proteins were separated by SDS-PAGE followed by Western blot analysis using anti-ERK½ **(A)**, anti-ERK ½ pT202/Y204 **(B)**, anti-nPKCϵ **(C)**, anti-PP2A **(D)**, and anti-DUSP9 **(E)**. Values represent n=3–7, mean +/- SD, expressed as the ratio of control MOCK group, which was considered 100%. Two groups were compared by unpaired two-tailed t-test, and four groups or more were compared by two-way ANOVA, where: p< 0.05 (*).

The effects of SMTNL1 on potential upstream regulators of insulin signaling were examined. Effector proteins of the insulin signaling pathway are regulated through a complex signaling cascade. MAPKs are significantly altered in insulin resistance and ERK1/2 regulates IRS-1 phosphorylation at Ser612. Thus, we investigated the expression and activity of ERK1/2 in MOCK and SMTNL1-transfected Ishikawa cells under control, P4, and GDB conditions. We evaluated the effects of SMTNL1 overexpression on ERK1/2 and the upstream regulator of ERK1/2 activity, nPKCϵ. No changes in ERK1/2 expression were detected under any experimental condition (**Figure 6A**). However, the activating phosphorylation of ERK1/2 at Thr202/Tyr204 increased by 184.33% (p=0.0337) and 158.67% (p=0.0369) in the MOCK GDB group compared with the control MOCK and P4 MOCK groups, respectively. The elevated ERK1/2 phosphorylation was attenuated by 153% (p=0.0451) in the FT-SMTNL1 GDB group compared to the MOCK GDB group (**Figure 6B**).

The expression of potent regulators of ERK1/2 activity was evaluated. Novel PKCϵ contributes to phosphorylation and activation of ERK1/2 MAPK by elevating ERK1/2 phosphorylation. Similarly to ERK1/2 activity, the GDB environment increased nPKCϵ activity by 46.125% (p=0.059) compared to the MOCK control, and nPKCϵ expression was reduced by 67.125% (p=0.0190) in response to SMTNL1 overexpression in GDB environment (**Figure 6C**). Further elements that contribute to the regulation of ERK1/2 by decreasing its activating phosphorylation site are protein phosphatase 2A (PP2A) and dual-specific phosphatase 9 (DUSP9). The expression of PP2A, a Ser/Thr protein phosphatase dephosphorylating ERK1/2, was elevated by 68.67% (p=0.0420) in response to P4. In addition, the PP2A level was increased by 131.33% (p=0.0180) in response to SMTNL1 overexpression in the GDB group compared to the GDB MOCK control group (**Figure 6D**). Comparing FT-SMTNL1 overexpressed groups the control group shown a decrement by 118.75% (p=0.0228) compared with the GDB SMTNL1 overexpressed group, regarding PP2A expression. Expression of the DUSP9, another potential regulator of ERK1/2, was enhanced by 346,66% (p=0.0104) in the GDB environment, and by 208,17% (p=0.0231) in P4 treatment upon SMTNL1 transfection (**Figure 6E**). Comparing the SMTNL1 overexpressed groups there was an elevation of DUSP9 expression by 279% (p=0.0443) comparing control and GDB samples. Our data suggests that SMTNL1 can attenuate the activity of ERK1/2 MAPK indirectly by decreasing the expression of nPKCϵ and increasing the expression of PP2A and DUSP9 in insulin-resistant endometrial epithelial cells.

To clarify that the MAPK pathway assumed by us really affects IRS-1, we used the selective inhibitor of ERK1/2, U0126. Based on our results, without any change in the expression of ERK1/2 (**Supplementary Figure S5A**), the phosphorylation of ERK1/2^pT202/Y204^ decreased under all conditions with U0126 inhibition (**Supplementary Figure S5B**). In parallel with the inhibition of ERK1/2, the phosphorylation of IRS-1^pS612^ decreased (**Supplementary Figures S5C, D**), and the glucose uptake measured by the NBGD assay increased in parallel (**Supplementary Figure S5E**), which clearly proves the insulin-sensitizing role of ERK1/2 inhibition.

### 3.7 SMTNL1 promotes progesterone-dependent glycogen storage and glucose uptake in endometrial epithelial cells

**Figure 7:**
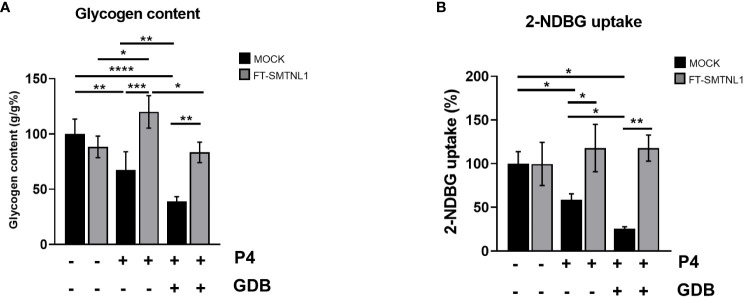
SMTNL1 facilitates glucose uptake and glycogen storage of Ishikawa cells. Glycogen content of control/P4/GDB treated Ishikawa cells were normalized to protein concentration. Control MOCK group value was taken as 100% and all other all other values were compared to that and expressed as ratio. Data are displayed as bar charts, values represent n=3-9 mean +/- SD. Groups were compared by two-tailed t-test. Bars represent n=3, mean +/- SD. p< 0.05 (*), p<0.01 (**), p<0.001 (***) and p<0.0001 (****) **(A)**. 2-NDBG glucose uptake of Ishikawa cells were assessed in control, progesterone (P4), and gestational diabetes (GDB) conditions with or without SMTNL1 overexpression. Values were normalized and expressed as the ratio of the control MOCK group, which was considered 100%. Bars represent n=3, mean +/- SD. Two groups were compared by unpaired two-tailed t-test, and four groups or more were compared by two-way ANOVA, where: p< 0.05 (*) and p<0.01 (**) **(B)**.

The requirement for excess glycogen storage is crucial for uterine endometrium homeostasis. SMTNL1 promotes glycogen synthesis and supports essential functional changes during endometrial differentiation. To validate the effects of SMTNL1 on glycogen storage, glucose metabolic status was assessed in Ishikawa cells in control, P4, and GDB conditions, with or without SMTNL1 overexpression (**Figure 7A**). SMTNL1 transfection significantly increased glycogen content in the P4 and GDB groups by 52,5% (p=0.0002) and by 44.55% (p=0.0024) compared to the corresponding MOCK controls, respectively. Glycogen storage was decreased in the P4 and GDB environments in Ishikawa cells by 32.5% (p=0.0091) and 61.25% (p<0.0001), in MOCK-transfected samples compared to untreated MOCK samples, respectively. GDB treatment reduced the glycogen content by 28.75% (p=0.01) compared to P4 MOCK. Comparing SMTNL1 overexpressing samples, a 36.67% (p=0.0226) decrease in glycogen content was detectable in GDB group compared to values of P4 treatment. The depletion of glycogen content in the P4 group corresponds to a physiological phenomenon; pregnant women also show mild signs of insulin resistance during pregnancy. The even more pronounced depletion of glycogen storage in the GDB group is due to the hyperglycemic-hyperinsulinemic environment. Glycogen depletion in both the P4 and GDB groups was restored by expressing FT-SMTNL1. Thus, SMTNL-1 exhibits a potent insulin-sensitizing effect in both the P4 and GDB conditions (**Figure 7A**).

To further evaluate the effects of SMTNL1 on glucose homeostasis in endometrial epithelial cells, 2-NDBG glucose uptake was measured. Ishikawa cells were incubated with 2-NDBG glucose for 1 hour, and the utilized glucose content was quantified by the 2-NDBG fluorescence intensity. SMTNL1 significantly increased glucose uptake in Ishikawa cells (**Figure 7B**) by 59.13% (p=0.0459) and 92.47% (p=0.0037) in the P4 and GDB environments, compared with their corresponding MOCK groups, respectively. The amount of utilized glucose decreased by 41.33% (p=0.0376) and 76.67% (p=0.0125) in the P4 MOCK and GDB MOCK transfected groups compared with the control MOCK group, respectively. GDB treated group show a decreased uptake of 2-NDBG glucose compared with P4 MOCK group by a decrement of 33.34% (p=0.0127). The downregulation of glucose uptake in response to P4 and GDB and the elevation of glucose uptake upon FT-SMTNL1 overexpression correlates with the glycogen storage results (**Figures 7A, B**), indicating that SMTNL1 sensitized the cells to insulin. Regarding expression of Glut4 transporter, neither of the applied treatments evoked significant change in the expression of the protein (**Supplementary Figure S4A**), hence the indicated change in the glycogen storage and the 2-NDBG uptake might be regarded to the altered localization of the transporter, which is caused by the FT-SMTNL1 overexpression.

## 4 Discussion

The importance of endometrial epithelial cells in the initial stages of embryo implantation and uterine growth has been well established. Endometrial cells provide the first contact site for blastocysts (21). Both the synchronized dialogue between epithelial cells and blastocyst and the homeostasis and differentiation of endometrial cells are crucial. Dysfunctional or defective epithelium may result in reproductive failure. However, the pathomechanisms of accompanying disorders, such as insulin resistance and gestational diabetes, are unclear. We hypothesized that one of the major orchestrating hormone-dependent proteins is SMTNL1. SMTNL1 KO animals exhibit a defective reproductive phenotype with high embryonal lethality, low embryo numbers after implantation, and longer intervals between pregnancies compared to WT littermates. In addition, SMTNL1 KO mice exhibit a gestational insulin-resistant phenotype, indicating crosstalk between signaling pathways regulated by SMTNL1.

In the present study, we used Ishikawa cells, a well-described cellular model of endometrial cells derived from a human endometrial epithelial adenocarcinoma (18, 22). Ishikawa cells represent the luminal epithelium on the surface of the uterine cavity and the glandular epithelium, which makes up the tubular glands and reaches deeply into the endometrial stroma (23). Control and MOCK transfected Ishikawa cells appear botryoidal, with polygonal and pleomorphic features, irregularly shaped nuclei, and small prominent nucleoli with high nuclei/cytoplasmic ratio, as observed in previous studies (23). The addition of progesterone triggers differentiation, with lower cell count and enlarged cells with small vacuoles and pleomorphic nuclei. The hyperglycemic state with progesterone prevented the morphological transformation of Ishikawa cells, and the cuboidal structure was maintained (**Figure 1**). This is in line with previous publications showing that changes in the luminal surface of rat endometrium in response to progesterone and estradiol are progressive, leading to the formation of dense microvilli and numerous droplets (24). Interestingly, the overexpression of SMTNL1 initiated the differentiation of endometrial cells, and these changes were more prominent in the presence of progesterone. Strikingly, elevated SMTNL1 rescued the differentiation arrest of Ishikawa cells in GDB environment and promoted morphological changes. Differentiation was assessed by measuring the gene expression of MUC-1, a differentiation marker. MUC-1 may also be a fertility and differentiation marker during the implantation period of the female cycle and its timely activation and inhibition is vital (1, 25) (**Supplementary Figure S6**).

To determine the mechanism for the cytoskeletal changes elicited by SMTNL1, SMTNL1-dependent enzymes regulating cell motility and shape were investigated. Our data suggests that SMTNL1 reduces the expression of MYPT1, the regulatory subunit of MP, in the presence of progesterone, and these changes are more prominent in the presence of progesterone and hyperglycemia (**Figure 2A**). MYPT1 expression decreased significantly in response to SMTNL1 overexpression in endometrial epithelial cells. This result is in line with a previous study showing that SMTNL1 downregulates MYPT1 gene expression in skeletal muscle and the smooth muscle of the thoracic aorta and uterus (15).

The major target of MP is non-muscle myosin, which plays crucial roles in processes requiring cellular remodeling, differentiation, movement, cell adhesion, cell migration, morphogenesis, and development via actin cross-linking and contractile functions (26). Based on our data and studies in murine endometrial tissues (27), we suggest that the role of MP is inevitable in endometrial epithelial cells and MYPT1 is the major isoform in the MP present in Ishikawa cells (28). In addition, SMTNL1 can inhibit MP activity in both smooth muscle (12) and endometrial epithelial cells by altering MYPT1 expression, which affects MLC20 phosphorylation (**Figures 2A**, **3C**).

SMTNL1 negatively influences the migration of endometrial epithelial cells; SMTNL1 overexpression decreases MP activity and increases the phosphorylation of MLC20 (**Figures 2A, C**, **3C**), especially in the P4 and GDB models. This finding is in agreement with a previous study showing that SMTNL1 overexpression in C2C12 myoblasts hampers cell migration, possibly through inhibition of MP (16). Moreover, inhibition of MP also reduced cell migration in HepG2 cells (15). MP may be involved in cell body contraction and tail detachment (29, 30). One suggested mechanism is a slower turnover rate of MLC20 phosphorylation in the cell center in response to the inhibition of MP, resulting in more stable adhesive structures and, hence, a slower migration rate. Overall, our results and previous studies indicate that MP facilitates Ishikawa cell migration, and cell migration is reduced in the insulin-resistant state by SMTNL1 (**Figure 4A**). This finding is important because an imbalance in steroid hormones can affect epithelial integrity and the migration capacity of endometrial cells, and numerous reports have shown a correlation between steroid hormone imbalance and metabolic disorders. The expression of the diabetes-obesity syndrome in C57BL/KsJ (*db/db*) mice is accompanied by progressive cellular atrophy and dysfunction of the female reproductive tract (31), characterized by a marked depression in uterine epithelial cellular integrity (32) and decreased responsiveness to ovarian steroid hormone therapy (32, 33). In addition, obesity and insulin resistance initiate endometrial cancer formation (5–7, 34, 35).

Several lines of evidence in isolated uterine cell cultures suggest that insulin ligands and insulin receptor activity affect cellular proliferation and differentiation of endometrial cells. The metabolic consequences of endometrial hyperglycemic/hyperinsulinemia are associated with reproductive dysfunction and tissue atrophy (7). Our findings show that SMTNL1 plays a detrimental role in the insulin-related regulation of endometrial cells. IRS-1 phosphorylation at Ser612 was upregulated in the progesterone and hyperglycemic models without affecting the expression of IRS-1 (**Figures 5A, B**). SMTNL1 overexpression decreased the phosphorylation at Ser612, in P4 and in the GDB model. Under insulin-resistant conditions, the shift from Tyr to Ser phosphorylation of IRS-1 prevent the binding of IRS-1 to PI3K, which accounts for insulin signaling defects (36). The phosphorylation of the Ser612 residue of IRS-1, located proximal to the regulatory Tyr residues, suppresses the insulin signaling pathway via ERK1/2 regulation. ERK1/2 activation plays a major role in maintaining insulin-induced PI3K-dependent InR (37).

In our model, ERK1/2 was phosphorylated after chronic insulin treatment and overexpression of SMTNL1 suppressed ERK1/2 activity, especially in the presence of progesterone (**Figure 6B**). In addition, IRS-1 Ser612 phosphorylation was reduced, likely due to SMTNL1-dependent reduction of ERK1/2 activity. We suggest that ERK1/2 exerts insulin-desensitizing effects in endometrial epithelial cells through Ser612 phosphorylation and the regulation of IRS-1, as chronic activation of ERK1/2 leads to the downregulation of GLUT4 expression and decreased insulin-induced glucose transport (38).

ERK1/2 gene expression did not change but ERK1/2 phosphorylation and, presumably, activity decreased in response to SMTNL1 overexpression. These changes are likely due to the altered expression of upstream regulators such as Ser/Thr kinases or phosphatases. PKC isoforms play a critical role in ERK1/2 regulation in the endometrium (39). PKCe expression was decreased in the skeletal muscle of SMTNL1 KO animals (13), and decreased PKCε expression suppressed ERK1/2 activity and IRS-1 Ser612 phosphorylation in C2C12 cells (14). However, PKCe expression was affected by SMTNL1 in GDB condition in Ishikawa cells (**Figure 6C**). Uterine MAP kinase kinases were not altered in a microarray analysis of pregnant s*mtnl1* KO mice compared to WT mice (13). Thus, we investigated protein phosphatases as potential regulators. Protein phosphatase 2A (PP2A), a Ser/Thr specific protein phosphatase, is a negative regulator of ERK1/2 (37), and the expression of the catalytic subunit of PP2A increased upon progesterone treatment but the increase was eliminated in the insulin resistance model (**Figure 6D**). Surprisingly, SMTNL1 overexpression dramatically increased the expression and presumably the activity of PP2A (**Figure 6D**). This result contradicts data in skeletal muscle where PP2A expression was not regulated by SMTNL1 (14). Endometrial cell progesterone sensitivity might be the key to this phenomenon.

We also investigated the expression of another potential regulator, DUSP9, that dephosphorylates and inactivates ERK1/2 (40, 41). DUSP9 is positively regulated by SMTNL1 (13). The expression of DUSP9 was independent of progesterone but SMTNL1 overexpression in the insulin-resistant state significantly increased DUSP expression (**Figure 6E**). This finding is in line with previous findings showing that DUSP9 can impede insulin resistance *in vivo* in the *ob/ob* murine model by lowering blood glucose levels via dephosphorylation and inactivation of ERK1/2 (42). DUSP9 expression can be differentially modulated in an insulin-sensitive model by both the type of diet and tissue (41, 43). Finally, the fact that the treatment with the selective inhibitor of ERK1/2, U0126, had a clear insulin-sensitizing effect on both IRS-1 phosphorylation and glucose uptake. It is in line with previous findings in T lymphocytes showing the induction of glucose metabolism upon U0126 (44). It supports our hypothesis that SMTNL1 induces insulin sensitization through the inhibition of ERK1/2 by modulating PP2A and DUSP9.

Glucose uptake and glycogen content in endometrial epithelial cells were significantly lower in the hyperglycemic model combined with progesterone (**Figures 7A, B**). These findings agree with a previous study in *db/db* mutant diabetic mice showing that elevated systemic glucose and insulin concentrations and lower glycogen levels correlated with the severity and specificity of progressive cellular changes in uterine epithelial cells (35). In addition, insulin, but not progesterone, directly regulates glycogen synthesis through canonical acute inactivation of GSKα/β and noncanonical stimulation of GYS2 transcription in endometrial epithelial cells (45). SMTNL1 promoted glucose uptake in a progesterone-dependent manner and increased glycogen content in Ishikawa cells, compensating for insulin resistance (**Figures 7A, B**). This phenomenon was previously described in insulin-resistant myotubules; SMTNL1-overexpression initiated glycolysis (14) and regulated hexokinase at the gene expression level (16).

Taken together, the results of the present study indicate that the disordered proliferation of endometrial cells, which can lead to the development of endometriosis, embryonal implantation disorders, and endometrial cancer, occurs in the insulin-resistant condition. SMTNL1 acts as a unique progesterone-dependent cytoskeletal and gene expression regulator to prevent or dampen insulin resistant defects. Thus, SMTNL1 is a promising therapeutic target for drug development. SMTNL1 enhances the differentiation of endometrial epithelial cells in a progesterone-dependent manner and prevents cellular transformation during insulin resistance. SMTNL1 regulates the phosphorylation of MLC20 through inhibition of myosin phosphatase to inhibit cell migration in the insulin-resistant state in the presence of progesterone. These effects may be relevant to the treatment of endometrial dysfunction in metabolic diseases, in the treatment of endometriosis, and in the control of metastasis during the early stages of endometrial tumors. SNTNL1 may also play a role in preventing insulin resistance by contributing to progesterone-dependent insulin sensitization via decreasing Ser phosphorylation of IRS. Therefore, SMTNL1 may be a feasible therapeutic target for progesterone-dependent inhibition of endometrial epithelial cells during hyperglycemia and insulin-sensitizing endometrium in gestational diabetes or other metabolic disorders.

## Data availability statement

The original contributions presented in the study are included in the article/**Supplementary Material**, further inquiries can be directed to the corresponding author/s.

## Author contributions

IK: Data curation, Formal analysis, Investigation, Software, Validation, Writing – original draft. ÁU: Investigation, Validation, Writing – review & editing. RK: Data curation, Investigation, Writing – review & editing. FS: Investigation, Visualization, Writing – original draft. EK: Visualization, Writing – review & editing. BL: Conceptualization, Formal analysis, Funding acquisition, Project administration, Resources, Supervision, Writing – original draft.

## Glossary

**Table:**

2-NBDG	2-N-7-Nitrobenz-2-oxa-1,3-diazol-4-yl-Amino-2-Deoxyglucose
Akt1	Protein kinase B
DAPI	4′, 6-diamidino-2-phenylindole
DMEM	Dulbecco’s Modified Eagle’s Medium
ERK ½	Extracellular signal-regulated kinase
FBS	Fetal Bovine Serum
GDB	gestational diabetes
GLUT4	Glucose transporter type 4
HCS	High Content Screening
IR	insulin resistance
IRS-1	Insulin Receptor Substrate 1
MAPK	mitogen-activated protein kinase
MLC20	20 kDa myosin light chain
MP	myosin phosphatase
MPA	medroxy-progesterone-acetate
mTOR	Mammalian target of rapamycin
MUC-1	Mucin 1
MYPT1	myosin phosphatase target subunit 1
NEAA	Non-Essential-Amino-Acids
nPKCϵ	novel type protein kinase C ϵ
OD	17-β-oestradiol
PBS	phosphate-buffered saline
PI3K	phosphatidyl-inositol-3-kinase
PIC	Phosphatase Inhibitor Cocktail
PMSF	phenylmethylsulfonyl fluoride
P4	progesterone
PRL	prolactin
RIPA	radioimmunoprecipitation assay buffer
SD	standard deviation
SDS	sodium dodecyl-sulfate
SDS-PAGE	Sodium dodecyl-sulfate polyacrylamide gel electrophoresis
SMTNL1	Smoothelin-like protein 1.
