# Supplementary material for: Smoothelin-like protein 1 promotes insulin sensitivity and modulates the contractile properties of endometrial epithelial cells with insulin resistance
Source: Front Endocrinol (Lausanne). 2024 May 31;15:1375771. doi: 10.3389/fendo.2024.1375771 (PMC11176479; doi:10.3389/fendo.2024.1375771)
Supplement: Supplementary file 1 [file DataSheet_1.docx]

SUPPLEMENTARY MATERIAL

Smoothelin-like protein 1 promotes insulin sensitivity and modulates the contractile properties of endometrial epithelial cells with insulin resistance.

**Ilka Keller^1^, Ádám Ungvári^1^, Richárd Kinter^1^, Fanni Szalmás^1^, Endre Kókai^1^, Beáta Lontay^1*^**

^1^Department of Medical Chemistry, Faculty of Medicine, University of Debrecen, Debrecen, Hungary

*** Correspondence:**Dr. Beáta Lontay

Department of Medical Chemistry, Faculty of Medicine

University of Debrecen,

H-4032 Debrecen, Egyetem tér 1. Hungary

Phone: +36-52-412345

e-mail: lontay@med.unideb.hu

**Keywords: endometrium, insulin resistance, insulin signaling, migration, gene expression**

**Conflicts of interest:** The authors have declared that no competing interests exist.

**Supplementary materials and methods**

**Acute insulin treatment of progesterone and hyperglycaemic/hyperinsulinaemic Ishikawa cells**

Ishikawa cells were cultured in control/P4/GDB environment for 72 hours as described previously. Cells were supplemented with 100 nM acute insulin for 30 minutes after 72 hours of treatment, and then cells were lysed and assessed by SDS-PAGE electrophoresis followed by Western Blot analysis as described in Protein Extraction, Western Blot Analysis section of Materials and Methods.

**Treatment of progesterone and hyperglycaemic/hyperinsulinaemic Ishikawa cells with the selective ERK1/2 inhibitor U0126**

Ishikawa cells were cultured in control/P4/GDB environment for 72 hours as described previously, with or without the addition of 2 µM U0126 ERK1/2 inhibitor (Sigma-Aldrich, St. Louis, MO, USA, Catalog number: 662009) .Cells were lysed and assessed by SDS-PAGE electrophoresis followed by Western Blot, or 2-NDBG uptake analysis as described in Protein Extraction, Western Blot Analysis and Glucose uptake assay section of Materials and Methods.

**Supplementary results**

MYPT1 phosphorylation on Thr696 residue was further verified by normalizing band intensity of the results with the phospho-antibody to GAPDH loading control (Figure S3A). Application of P4 treatment decreased the phosphorylation of MYPT1 by 49% (p=0.0399) compared to untreated MOCK control. This decrease was moderated by GDB treatment, where an elevation of 30.67% (p=0.0393) was detectable compared with P4 MOCK treated group. SMTNL1 overexpression elevated MYPT1 inhibitory phosphorylation by 13.6% (p=0.0419) and by 31.16% (p=0.0184) compared with untransfected P4 and GDB MOCK groups, respectively.

**Supplementary figures and tables**

**Figure S1. Effect of FT-SMTNL1 transfection on Ishikawa cells.** Control (no transfection), empty vector transfected (MOCK) and FT-SMTNL1 plasmid transfected (FT-SMTNL1) Ishikawa cells were examined (A-D, F-G). Immunofluorescent staining of FT-SMTNL1 overexpressed Ishikawa cells. Anti-Flag (A) and anti-SMTNL1 (D) antibodies were applied and detected by Alexa 488 fluorophore (green), and nuclei were stained with DAPI (blue). Fluorescent signals were detected using an automated high-content screening instrument. Scale bars: 20 μm Fluorophore Alexa 488 intensity labelling anti-Flag (C) and anti-SMTNL1 (F) primary antibodies have been measured, by built in intelligent software of high content screening instrument. Data were normalized to cell numbers obtained from nuclei number stained with DAPI. Control group value was taken as a 100%, and all other groups were expressed as ratios. Data was plotted as bar charts, where one bar represents n=3, mean +/- SD. Groups were compared using unpaired two-tailed t-test for comparison of two groups, and by one-way ANOVA, where *: p< 0.05, **: p<0.01. (B) Cell lysates were assessed by Western blot analysis using anti-Flag antibody. Values represent n=5, mean +/- SD. Bar charts display the expression levels of Flag, in untreated control, empty vector-transfected MOCK and FT-SMTNL1 overexpression groups. Control group expression was taken as 100%, and all other groups were compared to that using unpaired two-tailed t-test for comparison of two groups, and one-way ANOVA, where p< 0.05 (*) and p<0.0001 (***). (E) Cell lysates were assessed by Western blot analysis using anti-SMTNL1 antibody, bands were normalized to loading control GAPDH. Values were represented as bar charts, where each column represents n=3-5, mean +/- SD. Control group expression was taken as 100%, and all other groups were compared to that using unpaired two-tailed t-test, where p< 0.05 (*). (G) Effect of FT-SMTNL-1 overexpression on cell viability of Ishikawa cells was analyzed by Alamar Blue assay. Control group viability was taken as 100%, all other values were compared to that value, and expressed as ratios. Bars represent untreated control, empty vector treated MOCK and FT-SMTNL1 treated group viability %, n=3, mean +/- SD. Groups were compared using unpaired two-tailed t-test for comparison of two groups, and by one-way ANOVA.

**Figure S2. Demonstration of insulin resistance in the Ishikawa cell line** Ishikawa cells were maintained in control/P4/GDB environment with or without the addition of 30 min acute insulin treatment. Cell lysates were assessed by Western blot analysis using anti-Akt-1(A) and anti Akt-1^pS473^ (B) primary antibodies. Values were represented as bar charts, where each column represents n=3-6, mean +/- SD. Control group value was taken as 100%, and all other groups were compared to that using unpaired two-tailed t-test for comparison of two groups, and two-way ANOVA for comparison of four or more groups, where p< 0.05 (*).

**Figure S3. Phosphorylation of MYPT1 at the inhibitory Thr696 site normalized to GAPDH**

(A) Ishikawa cell lysates were assessed by Western blot analysis using anti-MYPT1^pT696,^ bands were normalized to loading control GAPDH. Values were represented as bar charts, where each column represents n=3-5, mean +/- SD. Control MOCK group expression was taken as 100%, and all other groups were compared to that using unpaired two-tailed t-test for comparison of two groups, and two-way ANOVA for comparison of four or more groups, where p< 0.05 (*).

**Figure S4.** Ishikawa cell lysates were assessed by Western blot analysis using anti-GLUT4 (A) primary antibody, bands were normalized to loading control GAPDH. Values were represented as bar charts, where each column represents n=3-5, mean +/- SD.

Figure S5. Selective inhibition of ERK1/2 improves insulin signaling and glucose uptake in insulin resistant Ischikawa cells

Ishikawa cells were cultured in control/P4/GDB environment for 72 hours with or without 2 µM U0126. Cell lysates were assessed by Western blot analysis using anti-ERK½ (A), anti-ERK ½^pT202/Y204^ (B), anti-IRS-1 (C) and anti-IRS-1^pS612^(D) antibodies. (E) Ishikawa cells were cultured in control/P4/GDB environments for 72 hours, with or without 2 µM U0126 treatment, and 2-NDBG uptake of cells were assessed. Values were represented as bar charts, where each column represents n=3-6, mean +/- SD. Control untreated group value was taken as 100%, and all other groups were compared to that using unpaired two-tailed t-test for comparison of two groups, and two-way ANOVA for comparison of four or more groups, where p< 0.05 (*), p< 0.005 (**) and p< 0.0001 (***).

**Figure S6. SMTNL1 promotes insulin sensitivity and regulates contractile function of Ishikawa cells on progesterone-dependent manner.** The graphical abstract illustrates that FT-SMTNL1 transfection evoked the insulin sensitivity of Ishikawa cells on a progesterone-dependent manner, meanwhile modulating key members of the insulin signaling pathway in Ishikawa cells Moreover FT-SMTNL1 was also able to promote differentiation and enhance contractile function of endometrial epithelial cells.

| ***Table S1 Antibodies used in Western Blot analysis*** | | | | | |  |
| --- | --- | --- | --- | --- | --- | --- |
| **Antibody** | **Producer** | **Catalog  Number** | **Type** | | **Dilution** | |
| IRS-1 | Cell Signalling Technology (Massachusetts, USA) | 2390 | Rabbit, monoclonal antibody | 1:1000 | | |
| Phospho-IRS-1 (Ser612) | Cell Signalling Technology (Massachusetts, USA) | 3193 | Rabbit, monoclonal antibody | 1:1000 | | |
| Akt-1 | Cell Signalling Technology (Massachusetts, USA) | 2938 | Rabbit, monoclonal antibody | 1:1000 | | |
| Phospho-Akt-1 (Ser473) | Cell Signalling Technology (Massachusetts, USA) | 3787 | Rabbit, monoclonal antibody | 1:1000 | | |
| Flag | Sigma–Aldrich (Missouri, USA) | F3165 | Rabbit, monoclonal antibody | 1:1000 | | |
| GAPDH | Sigma–Aldrich (Missouri, USA) | sc-47724 | Mouse Monoclonal antibody | 1:5000 | | |
| JNK | Cell Signalling Technology (Massachusetts, USA) | 9252S | Rabbit, monoclonal antibody | 1:1000 | | |
| Phospho-JNK(Thr138) | Cell Signalling Technology (Massachusetts, USA) | 81E11 | Rabbit, monoclonal antibody | 1:500 | | |
| ERK1/2 | Cell Signalling Technology (Massachusetts, USA) | 4695S | Rabbit, monoclonal antibody | 1:1000 | | |
| Phospho-ERK1/2 (Thr202) | Cell Signalling Technology (Massachusetts, USA) | 98168S | Rabbit, monoclonal antibody | 1:500 | | |
| MYPT-1 | BD Transduction Laboratories (New York, USA) | 612165 | Mouse Monoclonal antibody | 1:500 | | |
| Phospho-MYPT-1 (Thr696) | Merck-Millipore (Darmstadt, Germany) | ABS45 | Rabbit, monoclonal antibody | 1:500 | | |
| MLC20 | Cell Signalling Technology (Massachusetts, USA) | 3672S | Rabbit, monoclonal antibody | 1:500 | | |
| Phospho-MLC20 (Ser18) | Cell Signalling Technology (Massachusetts, USA) | 3674S | Rabbit, monoclonal antibody | 1:500 | | |
| PP2A | BD Transduction Laboratories (New York, USA) | 610555 | Mouse, monoclonal antibody | 1:1000 | | |
| nPKCε Antibody | Sigma–Aldrich (Missouri, USA) | sc-214 | Mouse Monoclonal antibody | 1:1000 | | |
| GLUT4 | Cell Signalling Technology (Massachusetts, USA) | 2213 | Mouse Monoclonal antibody | 1:500 | | |
| DUSP9 | Cell Signalling Technology (Massachusetts, USA) | 59277 | Rabbit, monoclonal antibody | 1:500 | | |

***Table S2 Primers used in RT-PCR analysis***

| **Gene** | **Direction** | | **Seqence 5’->3’** | **Concentration per reaction** | | **Tm** | | |
| --- | --- | --- | --- | --- | --- | --- | --- | --- |
| Human GAPDH | | Forward | AGCCTCAAGATCAGCAATG | | 0,4 µM | | 52,6°C |  |
| Human GAPDH | | Reverse | ATGGACTGTGGTCATGAGTCCTT | | 0,4 µM | | 57,9°C | |
| Human Cyclophillin A | | Forward | GTCTCCTTTGAGCTGTTTGCAGAC | | 0,4 µM | | 58,2°C | |
| Human Cyclophillin A | | Reverse | CTTGCCACCAGTGCCATTATG | | 0,4 µM | | 56,7°C | |
| Human MUC-1 | | Forward | TGCCGCCGAAAGAACTACG | | 0,4 µM | | 54,3°C | |
| Human MUC-1 | | Reverse | TGGGGTACTCGCTCATAGGAT | | 0,4 µM | | 56,2°C | |
