# Supplementary figures and images for: Smoothelin-like protein 1 promotes insulin sensitivity and modulates the contractile properties of endometrial epithelial cells with insulin resistance
Source: Front Endocrinol (Lausanne). 2024 May 31;15:1375771. doi: 10.3389/fendo.2024.1375771 (PMC11176479; doi:10.3389/fendo.2024.1375771)

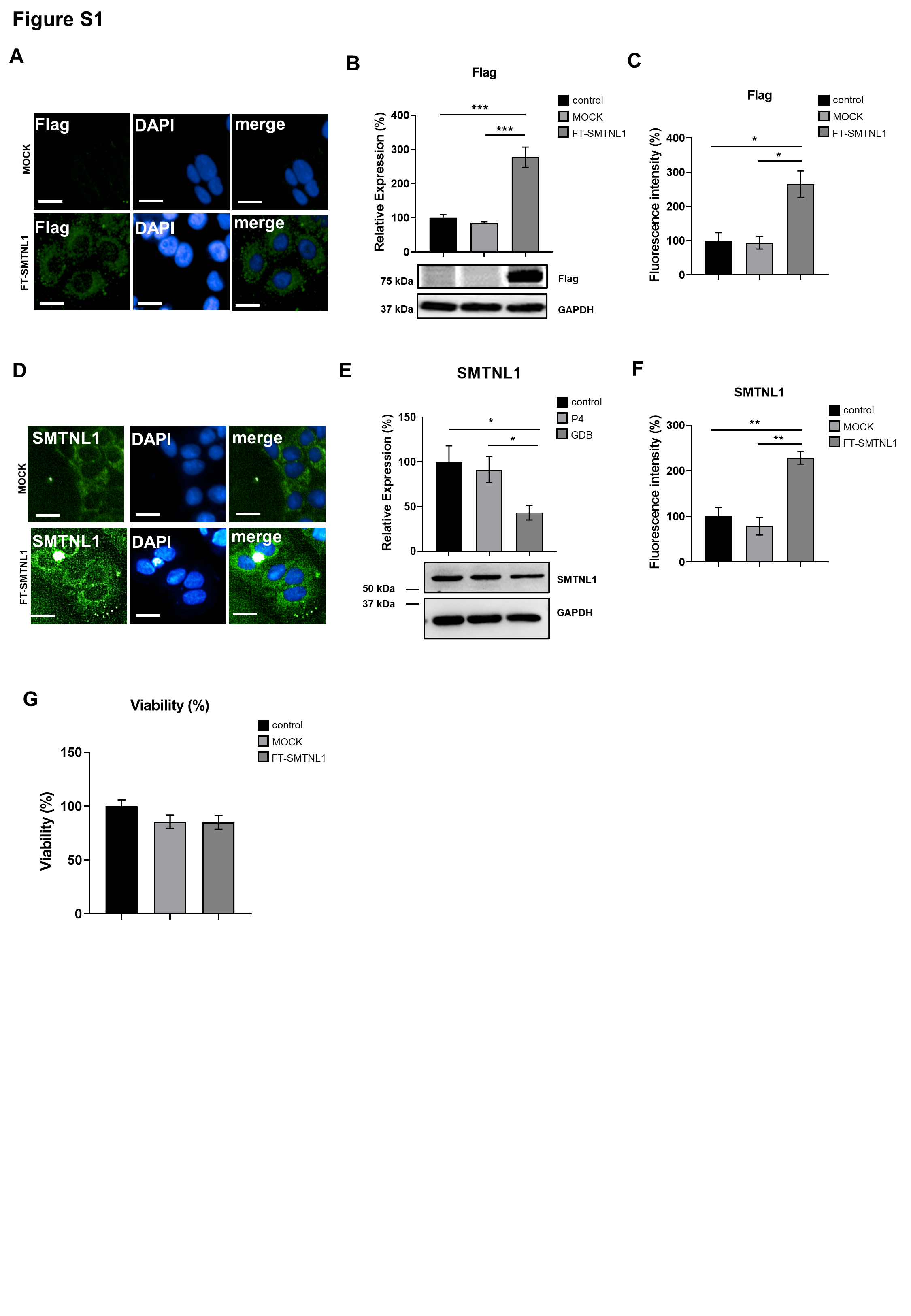

Supplement: Supplementary file 2 [file Image_1.tif]

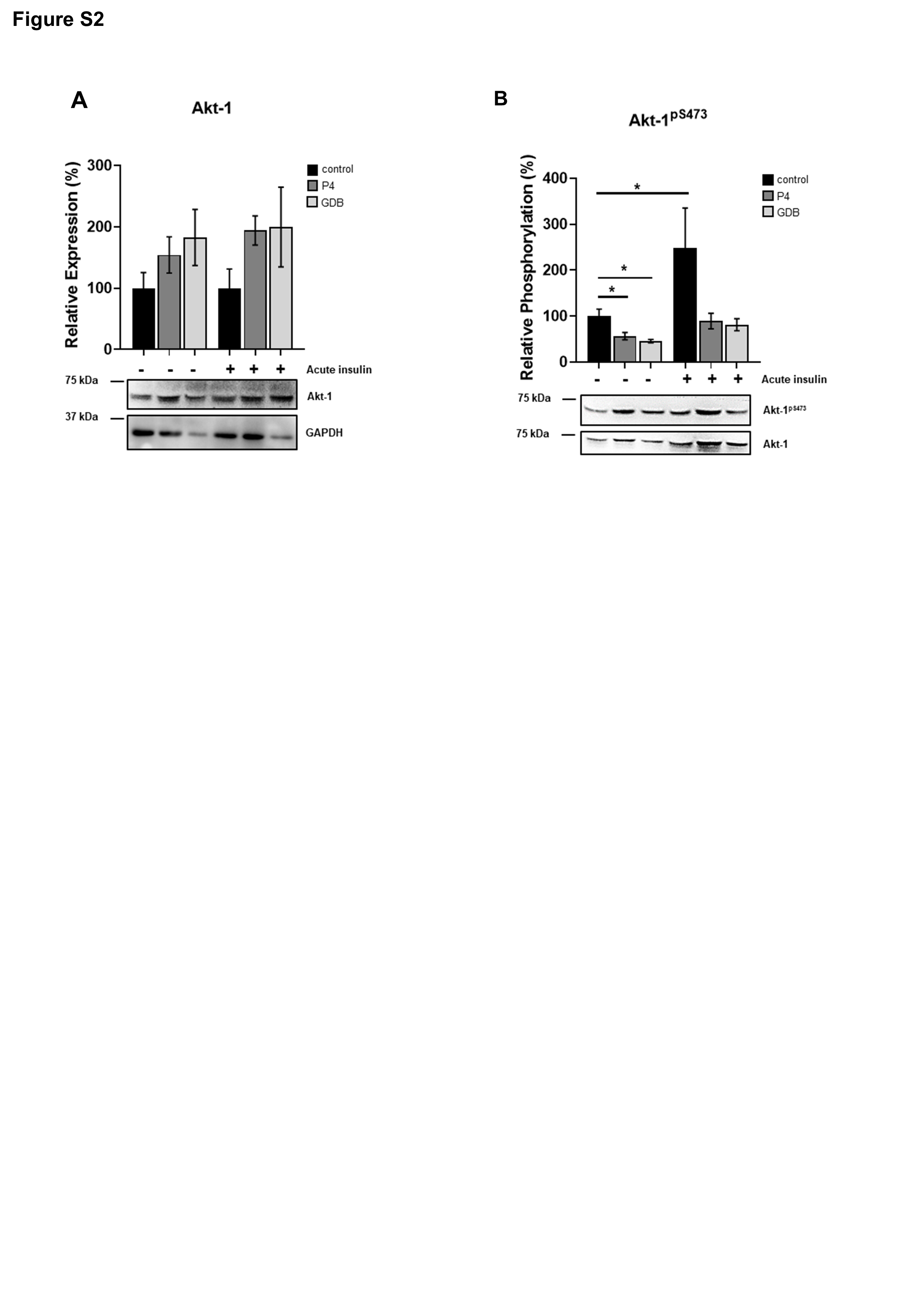

Supplement: Supplementary file 3 [file Image_2.tif]

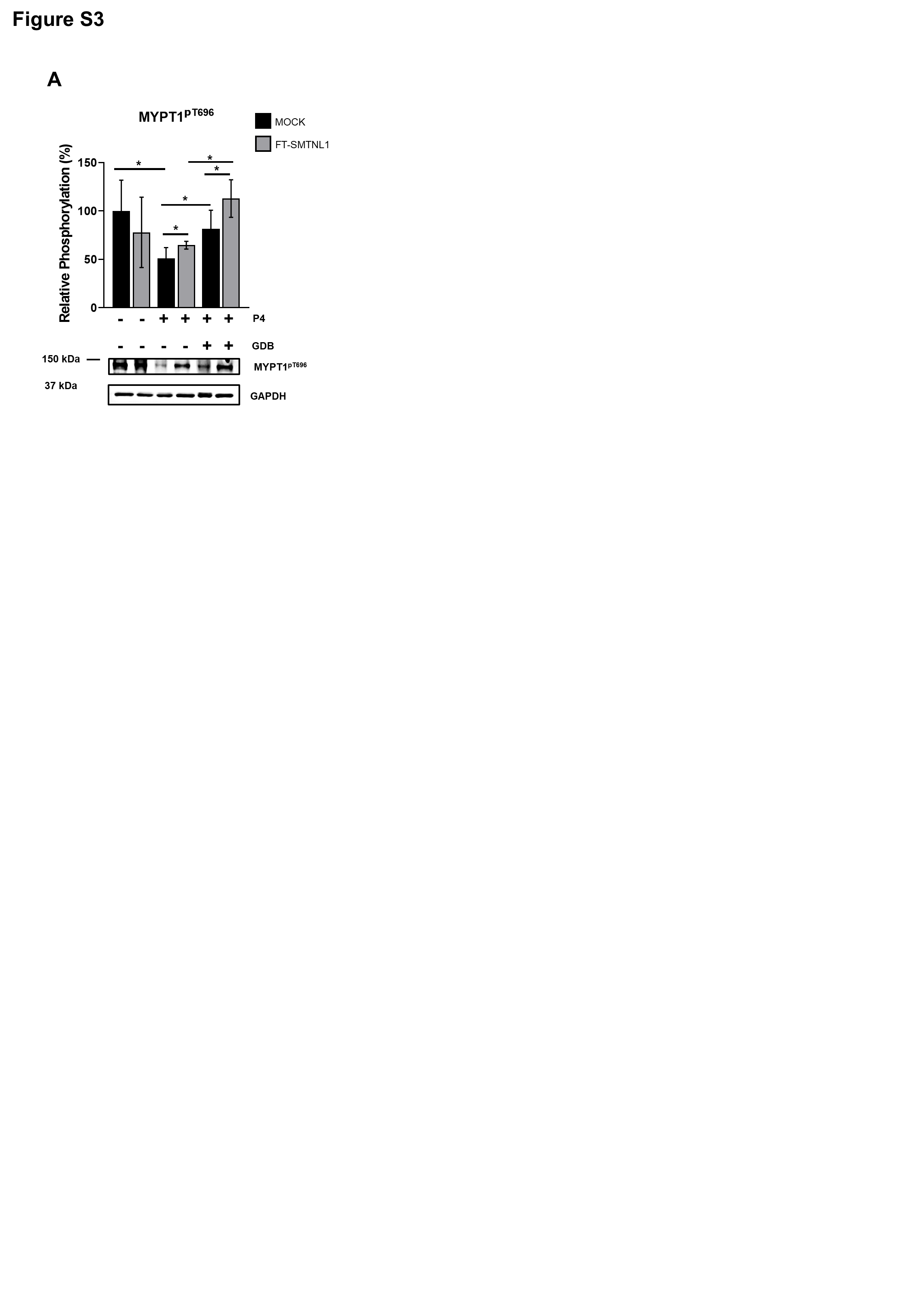

Supplement: Supplementary file 4 [file Image_3.tif]

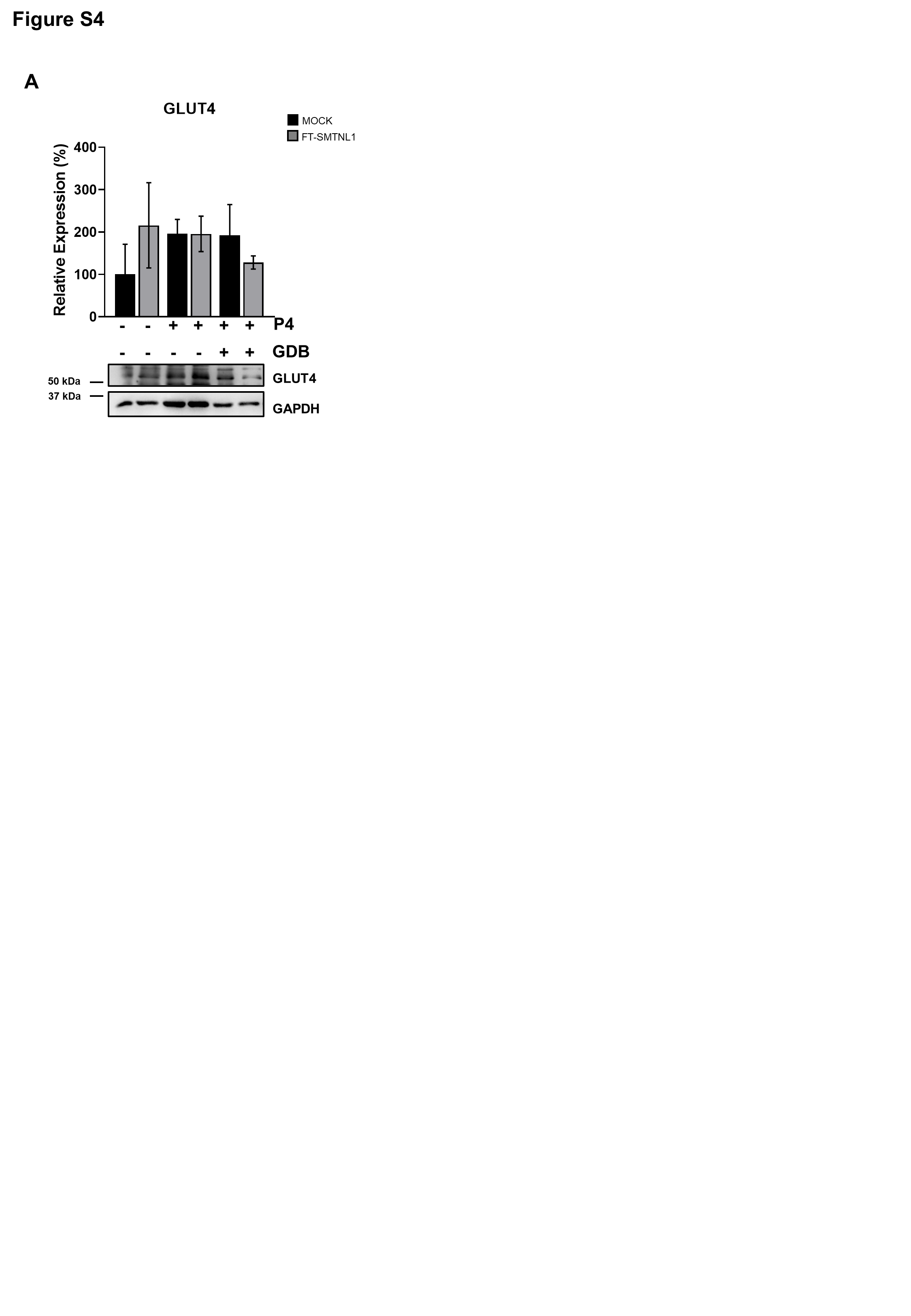

Supplement: Supplementary file 5 [file Image_4.tif]

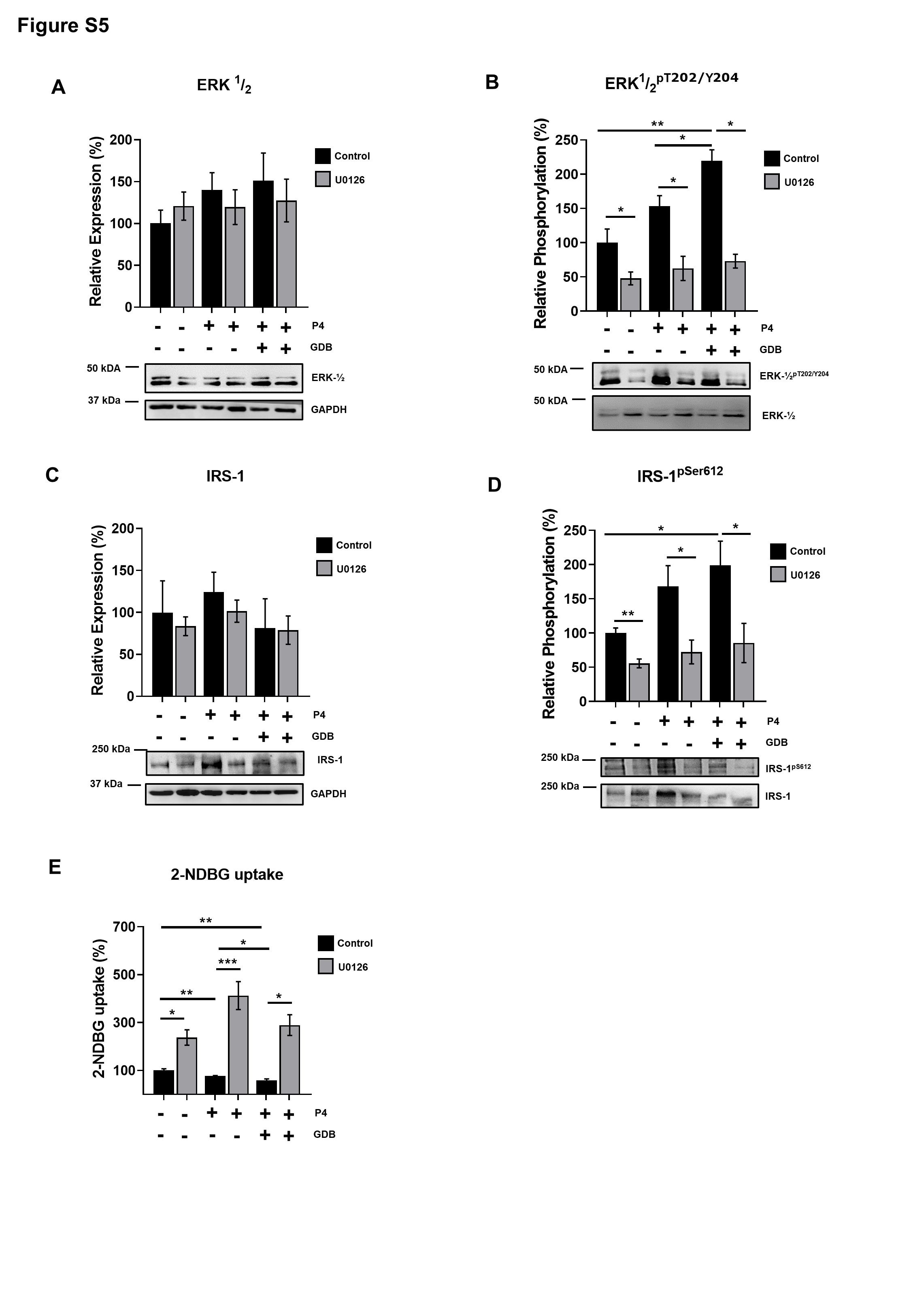

Supplement: Supplementary file 6 [file Image_5.tif]

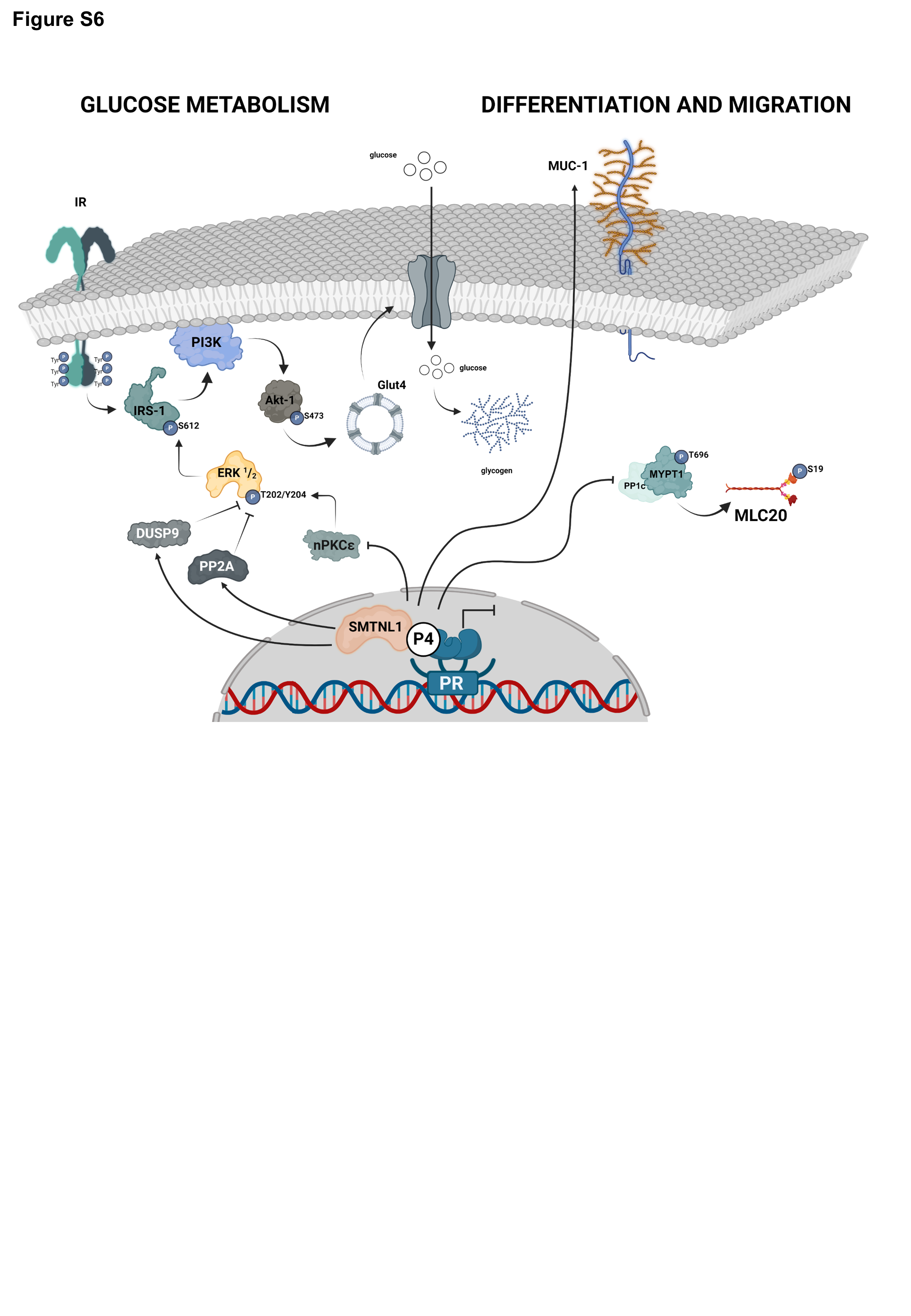

Supplement: Supplementary file 7 [file Image_6.tif]
